# Peptidyl ω-Asp Selenoesters Enable Efficient Synthesis of *N*-Linked Glycopeptides

**DOI:** 10.3389/fchem.2020.00396

**Published:** 2020-05-05

**Authors:** Jing-Jing Du, Lian Zhang, Xiao-Fei Gao, Hui Sun, Jun Guo

**Affiliations:** ^1^Key Laboratory of Pesticide and Chemical Biology of Ministry of Education, Hubei International Scientific and Technological Cooperation Base of Pesticide and Green Synthesis, International Joint Research Center for Intelligent Biosensing Technology and Health, College of Chemistry, Central China Normal University, Wuhan, China; ^2^Jiangxi Key Laboratory for Mass Spectrometry and Instrumentation, East China University of Technology, Nanchang, China; ^3^Hubei Key Laboratory of Cell Homeostasis, Hubei Province Key Laboratory of Allergy and Immunology, Key Laboratory of Combinatorial Biosynthesis and Drug Discovery, College of Life Sciences, Ministry of Education, Wuhan University, Wuhan, China

**Keywords:** *N*-linked glycopeptide, glycosylation, selenoester, aminolysis, chemical synthesis

## Abstract

Chemical synthesis is an attractive approach allows for the assembly of homogeneous complex *N*-linked glycopeptides and glycoproteins, but the limited coupling efficiency between glycans and peptides hampered the synthesis and research in the related field. Herein we developed an alternative glycosylation to construct *N*-linked glycopeptide via efficient selenoester-assisted aminolysis, which employs the peptidyl ω-asparagine selenoester and unprotected glycosylamine to perform rapid amide-bond ligation. This glycosylation strategy is highly compatible with the free carboxylic acids and hydroxyl groups of peptides and carbohydrates, and readily available for the assembly of structure-defined homogeneous *N*-linked glycopeptides, such as segments derived from glycoprotein EPO and IL-5.

## Introduction

Many proteins undergo co- or post-translational modifications, including phosphorylation, acetylation, and glycosylation to fulfill their functions (Walsh and Jefferis, 2006; Carubbi et al., 2019). It is estimated that glycosylation modifications are associated with approximately 50% of human proteins (Clerc et al., 2016; Oliveira-Ferrer et al., 2017) and 30% of approved biopharmaceutical proteins (Zou et al., 2020), which are critical for important biological processes in living systems, such as cell's adhesion, recognition, targeting, and differentiation (Varki, 2017; Bhat et al., 2019). Despite the importance of glycosylations, rigorous evaluation of the relationship between the precise structure and biological function of glycoproteins is complicated by the structural heterogeneity of the oligosaccharides in biological organisms, and the difficulty to obtain sufficient amounts of structure-defined glycoproteins with single glycoform from natural sources (Park et al., 2009).

**Scheme 1:**
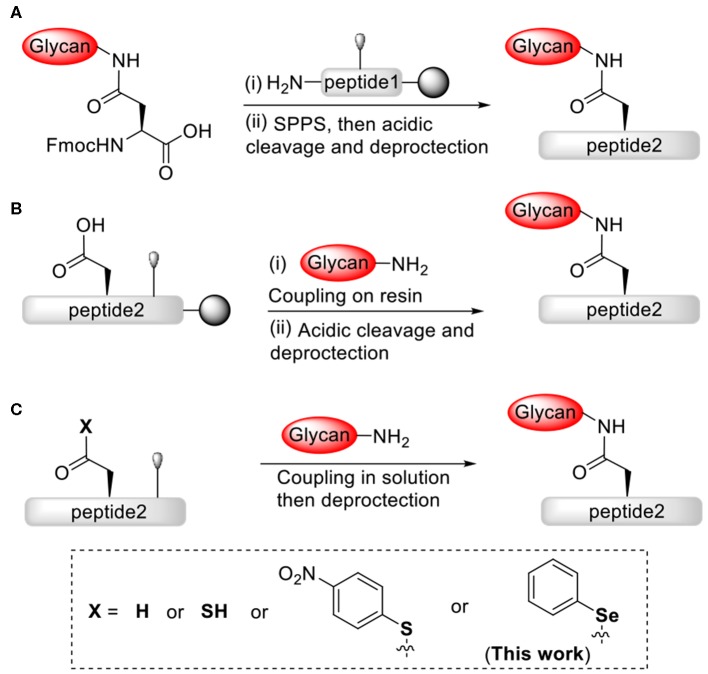
Effective strategies for chemical construction of *N*-linked glycopeptides: **(A)** stepwise strategy via solid-phase; **(B)** convergent strategy via solid-phase; **(C)** convergent strategy via solution phase.

In order to develop viable and efficient strategies to chemically construct homogeneous complex *N*-linked glycopeptides and glycoproteins, extensive efforts and advances have been made in the field (Payne and Wong, 2010; Wilson et al., 2013; Okamoto et al., 2014a; Wang and Amin, 2014; Fairbanks, 2019; Li et al., 2019), such as the resin-bound glycosylation (Kunz and Unverzagt, 1988; Vetter et al., 1995; Offer et al., 1996; Mezzato et al., 2005; Kajihara et al., 2006; Yamamoto et al., 2008; Piontek et al., 2009a,b; Chen and Tolbert, 2010; Conroy et al., 2010; Ullmann et al., 2012; Okamoto et al., 2014b; Reif et al., 2014; Lee et al., 2016; Schöwe et al., 2019) and solution glycosylation (Anisfeld and Lansbury, 1990; Cohen-Anisfeld and Lansbury, 1993; Kaneshiro and Michael, 2006; Wang et al., 2011, 2012, 2013; Aussedat et al., 2012; Nagorny et al., 2012; Sakamoto et al., 2012; Joseph et al., 2013; Chai et al., 2016; Schöwe et al., 2019). However, unneglectable limitations still remain in these strategies. Consumption of large amount of precious materials and low coupling yields usually occurred for the glycopeptide assembly on-resin via either the stepwise (Scheme 1A) or the convergent (Scheme 1B) strategy. Based on the aspartylation technology pioneered by Lansbury and co-workers (Scheme 1C) (Anisfeld and Lansbury, 1990; Cohen-Anisfeld and Lansbury, 1993), Danishefsky group and Unverzagt group developed the synthetic methods and optimized the pseudoproline dipeptide building block to construct the peptide fragement at the site of Asn-Xaa-Ser/Thr, and this approach significantly suppressed the formation of aspartimide byproducts during glycosylation (Ullmann et al., 2012; Wang et al., 2012). Although useful, requirement for additional metal catalysts or protected *C*-terminal carboxylic acid derivatives may limit the application of this strategy in glycopeptide assembly.

Notwithstanding substantial advances have been made in *N*-linked glycopeptides and glycoproteins synthesis, it is still a great challenge to efficiently achieve large *N*-linked gylcoproteins bearing complex glycan forms. The desired synthetic methods will have fewer protecting groups and modifications on the peptide and glycan fragments, and promote efficient and selective ligation reactions between fragments. Previously, our research group has developed a strategy for the convergent synthesis of *N*-linked glycopeptides via peptidyl ω-Asp *p*-nitrophenyl thioester-assisted glycosylation (Scheme 1C) (Du et al., 2016). This convergent strategy with direct aminolysis provides an access to complex *N*-linked glycopeptides, usually with good yields and simple operation, and is worthy of further investigating more reactions and applications.

Many investigators have proved that coupling of peptide fragments via direct aminolysis is a feasible method for preparation of peptides and glycopeptides. This method employs direct coupling reaction between peptide fragments bearing *N*-terminal free amines and peptide fragments bearing *C*-terminal active esters, such as oxoesters (Kemp and Vellaccio, 1975; Wan et al., 2008; Li et al., 2010), thioesters (Payne et al., 2008; Agrigento et al., 2014; LingáTung and Clarence, 2015; Gui et al., 2016) or selenoester derivatives (Grieco et al., 1981; Mitchell et al., 2015; Raj et al., 2015; Takei et al., 2017; Temperini et al., 2017; Du et al., 2018; Sayers et al., 2018a,b; Chisholm et al., 2020; Wang et al., 2020), eliminates the need for *N*-terminal cysteine residues or thiol ligation auxiliaries, which are generally required for the sequential native chemical ligation (Dawson et al., 1994; Kent, 2009). Notably, the active selenoesters or derivatives always offer enhanced reactivity compared to the thio- or oxoesters (Mitchell et al., 2015; Raj et al., 2015; Takei et al., 2017). Our previous studies have shown that the aminolysis of peptidyl selenoester is an efficient strategy for peptide and glycopeptide assembly (Yin et al., 2016; Du et al., 2018). Herein we are interested in pursuing a highly reactive peptidyl ω-Asp selenoester-assisted glycosylation methodology for constructing *N*-linked glycopeptides without coupling reagents (Scheme 1C). This methodology is assumed to be compatible with free carboxylic groups and hydroxyl groups of peptides and glycans.

## Results and Discussion

### Evaluation of the Reactivity of the Active Esters for Glycosidic Amide Bond Formation

**Figure 1:**
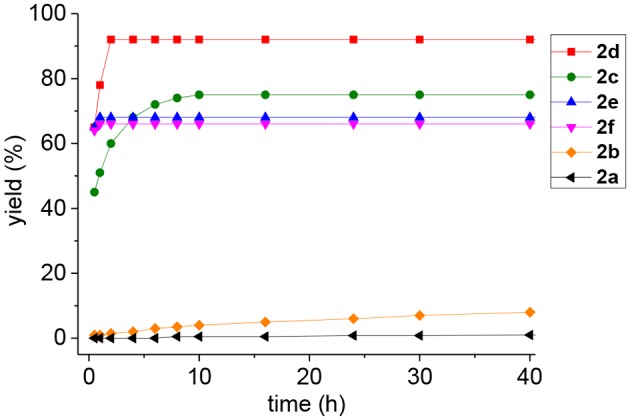
Reaction curves (yields of **3** vs. time) of the glycosidic linkage formation between **2a−2f** and **1a**.

To evaluate the methods for synthesizing *N*-linked glycopeptide synthesis via active ester-assisted aminolysis (Du et al., 2016), the activity and efficiency of different active esters were compared and investigated using model reactions, in which Fmoc-Gly ester **2** and glycosylamine **1a** (Likhosherstov et al., 1986; Cohen-Anisfeld and Lansbury, 1993) were condensed in DMSO to form β-anomer product **3** and monitored by HPLC (Table 1, Figure 1).

**Table 1:** Optimization of the active esters for the glycosylation reaction^a^.

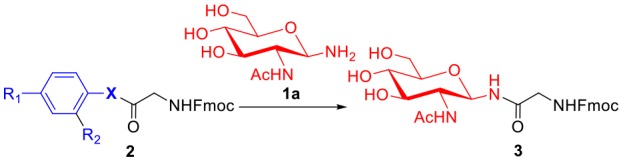						
**Entry**	**Ester**	**X**	**R_**1**_**	**R_**2**_**	**Time (h)^b^**	**Yield (%)^c^**
1	**2a**	O	H	H	>40	<1
2	**2b**	S	H	H	>40	8
3	**2c**	S	NO_2_	H	10	75
4	**2d**	Se	H	H	2	92
5	**2e**	Se	H	CHO	1	69
6	**2f**	Se	CHO	H	1	67

a *Reaction conditions: **1a** (10 μmol), esters (5 μmol) and DIPEA (10 μmol) in 1 mL of DMSO, rt*.

b *Consumption of >95% of the starting ester in glycosylation reaction was determined by HPLC*.

c *Determined by HPLC at 40 h*.

For oxoester **2a**, it has the lowest activity and almost no product was observed (Table 1, entry 1). For thioesters (Table 1, entries 2-3), phenyl thioester **2b** underwent glycosidic bond formation slightly faster than the oxoester **2a**, but it is not efficient enough to be applied in the *N*-linked glycopeptide synthesis; *p*-nitrophenyl thioester **2c** with a strong electron-withdrawing group reacts more efficiently, providing the target product in a yield of 75% within 10 h, which is consistent with previous studies (Hondal et al., 2001; Du et al., 2016). Therefore, the peptidyl *p*-nitrophenyl thioester has been successfully utilized to prepare *N*-linked glycopeptide in our lab (Du et al., 2016).

To improve the efficiency of glycosylation reaction, various selenoesters were assessed under the same conditions (Table 1, entries 4–6). For seleno-phenyl ester **2d**, it underwent complete conversion within 2 h, and afforded the target product **3** in 92% yield; for seleno-benzaldehyde esters **2e** with the *o*-benzaldehyde group and **2f** with the *p*-benzaldehyde group (Raj et al., 2015), they underwent complete conversion in <1 h, and gave the products in yield of 69 and 67%, respectively. We postulate that the participation of *o*-benzaldehyde (neighbor-participating group) and *p*-benzaldehyde, which both have electron-withdrawing groups can increase the phenyl selenoester's electrophile reaction rate, but also facilitate the hydrolysis reaction and reduce the yield of aminolysis product. Therefore, the seleno-phenyl ester **2d** affords an optimal balance between high reactivity and sufficient stability, will be appropriate for the selenoester-assisted aminolysis in glycosylation reactions.

As shown in Table 2, we compared the reaction kinetic data *p*-nitrophenyl thioester **2c** and seleno-phenyl ester **2d**. As expected, the glycosylation reaction for the product **3** between glycine-derived ester and glycosylamine follows a second-order kinetics, with a rate constant 0.0071 ± 0.0004 M^−1^ s^−1^ for **2c** and 0.0420 ± 0.0012 M^−1^ s^−1^ for **2d**, respectively. The seleno-phenyl ester is roughly 6-times faster than the *p*-nitrophenyl thioester to form the glycosidic amide bond.

**Table 2:** Kinetic studies for glycosidic bond formation^a^.

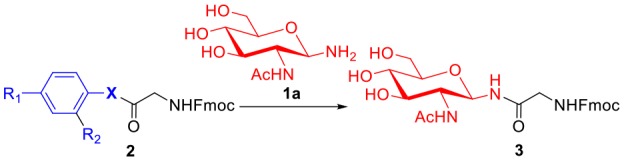					
**Ester**	**X**	**R**_**1**_	**R**_**2**_	***K*** **[M**^**−1**^**s**^**−1**^**]**	**Relative rates**
**2c**	S	NO_2_	H	0.0071 ± 0.0004	1
**2d**	Se	H	H	0.0420 ± 0.0012	6

a *Reaction conditions: **1a** (10 μmol), esters (5 μmol) and DIPEA (10 μmol) in 1 mL of DMSO, rt*.

### Condition Optimization

As depicted in Table 3, various glycosylation reaction conditions were evaluated for further optimization. From the results of optimizing the solvent (Table 3, entries 1–4), the efficiency of the glycosylation reaction was shown to be greatly boosted in DMSO, but the aqueous solution of NMP/PB is prone to decompose the seleno-phenyl ester **2d**. The amounts of DIPEA from 0.1 to 3.0 equivalents didn't significantly influence the yields (Table 3, entries 4–7). Additionally, we found that the product **3** was achieved in optimal yield when seleno-phenyl ester **2d** was treated with 2.0 equivalents of glycosylamine **1a** (Table 3, entries 4, 8–9). In order to maximize the glycosylation and minimize the hydrolysis, we selected the optimum conditions, i.e., 2.0 equivalents of DIPEA and glycosylamine **1a**, and 1.0 equivalent of seleno-phenyl ester **2d** were dissolved in DMSO.

**Table 3:** Reaction optimization and control experiments^a^.

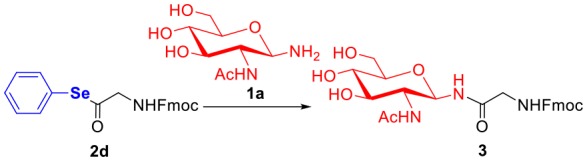					
**Entry**	**1a (equiv.)**	**2d (equiv.)**	**Solvent**	**DIPEA (equiv.)**	**Yield (%)^b^**
1	2	1	NMP/PB	2	52
2	2	1	NMP	2	78
3	2	1	DMF	2	76
4	2	1	DMSO	2	92
5	2	1	DMSO	3	90
6	2	1	DMSO	1	88
7	2	1	DMSO	0.1	85
8	1	1	DMSO	2	70
9	3	1	DMSO	2	93

a *Reaction conditions: **1a** (5-15 μmol), ester **2d** (5 μmol) and DIPEA (10 μmol) in 1mL of solvent, rt*.

b *Determined by HPLC at 2 h. PB = phosphate buffer (pH 7.4, 0.2 M)*.

### Substrate Scope

**Figure 2:**
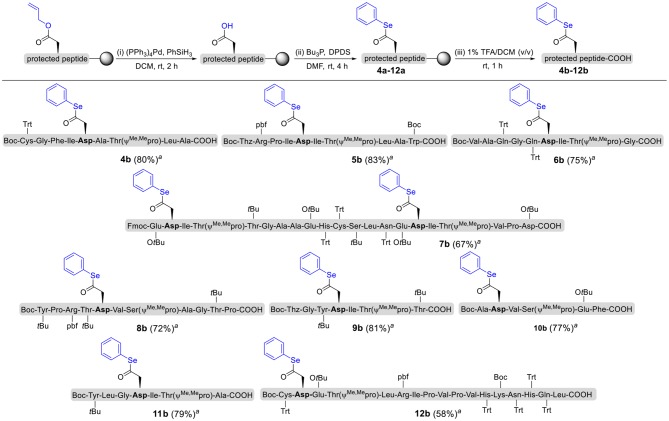
Solid-phase synthesis of peptidyl selenoesters **4b-12b**.

**Figure 3:**
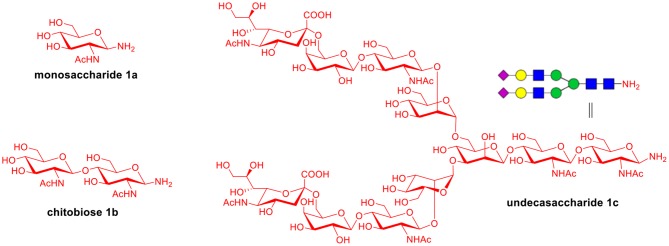
Structures of glycosylamines **1a-1c**.

To explore the universal applicability of selenoester-assisted glycosylation, we embarked on the attachment of seleno-phenyl esters to a series of peptides to assemble peptidyl ω-Asp selenoester substrates, and examined substrates that incorporating the free *C*-terminal carboxylic groups and unprotected glycosylamines. A series of partially protected peptides bearing selenoesters at the ω-aspartyl terminus (including pseudoproline dipeptides that suppress aspartimide formation) were successfully prepared for evaluation (Ullmann et al., 2012; Wang et al., 2012). These peptide substrates were conducted via stepwise solid-phase peptide synthesis (SPPS), the general synthetic procedures for **4b-12b** are outlined in Figure 2 (more details are shown in the Supporting Information). The installation of phenyl selenoester group at the ω-aspartyl terminus is straightforward on the resin: firstly, the allyl esters were removed; subsequently, the ω-aspartyl carboxyl groups were converted to selenoesters (**4a-12a**); finally, these peptidyl selenoesters were cleaved from the resin. The ω-aspartyl selenoester peptide substrates (**4b-12b**) were isolated via reverse-phase HPLC purification in 58–83% yields. In addition, the glycosylamines (Figure 3) for the study are monosaccharide **1a**, chitobiose **1b** and undecasaccharide **1c** (extracted from fresh egg yolks) (Seko et al., 1997; Sun et al., 2014).

With peptidyl selenoesters and glycosylamines in hand, the glycosylation reactions at the site of natural ω-asparagine linkage were evaluated. On the one hand, the coupling of monosaccharide **1a** and peptides **4b-6b** gave glycosylated peptides **4c-6c** in approximately 69%-83% isolated yields (Table 4, entries 1–3), proving the feasibility of utilizing unprotected glycosylamines together with peptidyl selenoesters bearing free *C*-terminal carboxylic groups in glycosylation reactions. To our delight, peptide **7b** containing two ω-asparagine selenoesters, still gave an isolated yield of 80% of product **7c** derived from multiply glycosylated protein erythropoietin (EPO; fragment 22–43) (Park et al., 2009; Wang et al., 2013; Wilson et al., 2013) with two glycosylation modifications (Table 4, entry 4). On the other hand, this strategy also afforded good results for glycosylation of disaccharides. As shown in entries 5–7, coupling of chitobiose **1b** and peptidyl selenoesters **5b**, **8b**, and **9b** formed glycosidic bond at ω-asparagine residue with excellent yields.

**Table 4:** Scope of the peptidyl selenoester-based glycosylation^a^.

**Entry**	**Peptide + glycan ratio (P:G)**	**Product**	**Isolated yield**
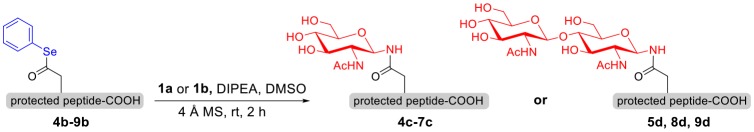			
1	**4b** **+** **1a** (1:2)	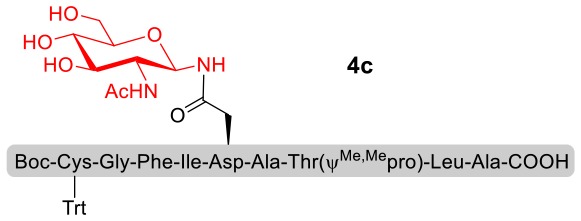	69%
2	**5b** **+** **1a** (1:2)	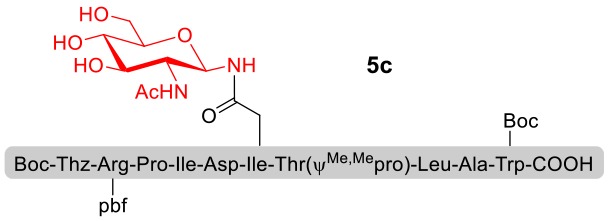	83%
3	**6b** **+** **1a** (1:2)	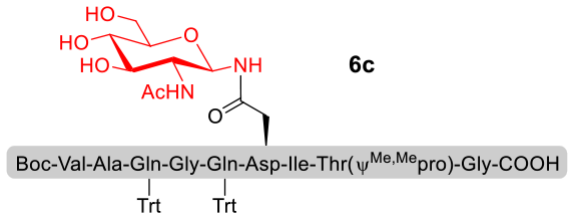	78%
4	**7b** **+** **1a** (1:2)	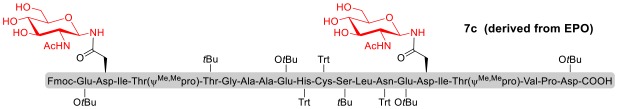	80%
5	**5b** **+** **1b** (1:2)	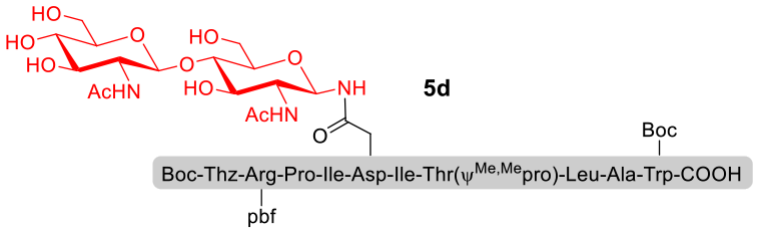	79%
6	**8b** **+** **1b** (1:2)	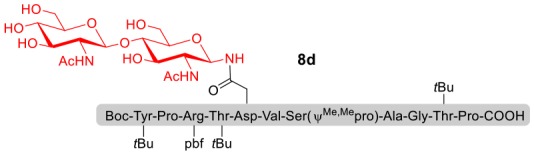	82%
7	**9b** **+** **1b** (1:2)	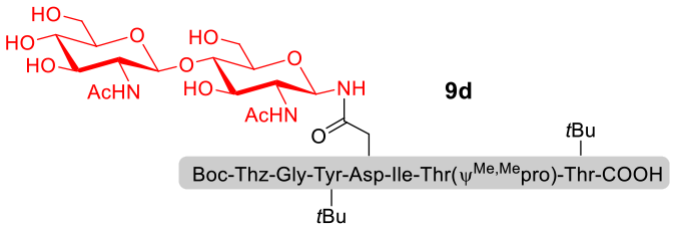	84%

a *Reaction conditions: **1a** (10 μmol), selenoester peptides (5 μmol) and DIPEA (10 μmol) in 1 mL of DMSO, rt, 2 h*.

For this methodology, it is noteworthy that the desired *N*-linked glycopeptides are synthesized rapidly only through mixing two substrates, without using a condensation reagent, and the workup procedure is simple. Excitingly, the free carboxylic groups of ω-aspartyl peptide segments were readily converted into peptidyl selenoesters for further condensation with various glycosylamines. Additionally, each amino acid protecting group in glycopeptide can be easily removed in an acidic environment.

### Syntheses of *N*-Linked Glycopeptides With Complex-Type Oligosaccharide

As shown in Table 5, the protocol of selenoester-mediated glycopeptide synthesis is extended to complex-type oligosaccharide amines. Given the structural complexity of the precious undecasaccharide amine **1c**, an excessive amount of peptidyl selenoester (1.5:1) was used, and the final products (**10e**, **11e**, **12e**) of the peptides modified with undecasaccharides were achieved in good yields of 59–65% (Table 5, entries 1–3). Specially, product **12e** corresponds to the truncated segment of the glycoprotein found in human interleukin-5 (IL-5, an eosinophil chemotactic factor, fragment 26–43) (Coffman et al., 1989; Liu and Dong, 2018).

**Table 5:** Selenoester-mediated glycosylation^a^.

**Entry**	**Peptide + glycan ratio (P:G)**	**Product**	**Isolated yield**
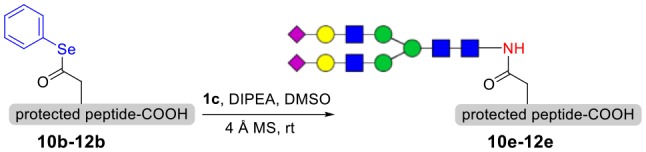			
1	**10b** **+** **1c** (1:1.5)	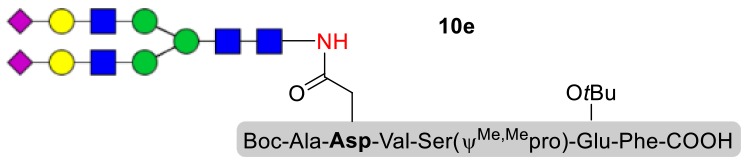	65%
2	**11b** **+** **1c** (1:1.5)	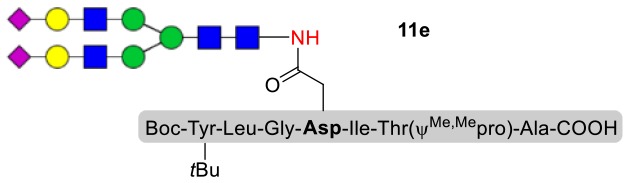	59%
3	**12b** **+** **1c** (1:1.5)	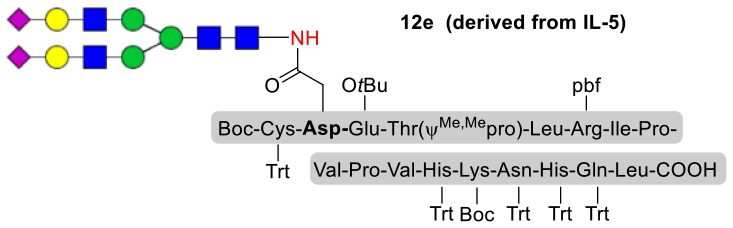	62%

a *Reaction conditions: **1c** (3 μmol), **10b-12b** (2 μmol), DIPEA (4 μmol) in 0.5 mL of DMSO, 4 Å MS, rt, 6 h*.

## Conclusion

In this work we have developed a convergent and facile synthetic methodology to construct homogeneous *N*-linked glycopeptides from the peptides with ω-Asp phenyl selenoester, the use of peptidyl selenoesters has the merits of simple operation and obtained excellent yields of *N*-linked glycopeptides, such as truncated segments derived from glycoprotein EPO or IL-5. This selenoester-mediated glycosylation provides several advantages: the reactivity of the peptide ester is improved, the complex sialyloligosaccharide in its native form without protection, it is not only compatible with free *C*-terminal carboxylic acid groups, but also rapidly forms glycosidic bond without additional coupling reagents or catalysts. This method will be further applied to the formation of homogenous *N*-linked glycopeptides and glycoproteins with therapeutic potential.

## Data Availability Statement

The raw data supporting the conclusions of this article will be made available by the authors, without undue reservation, to any qualified researcher.

## Author Contributions

JG conceived the project. J-JD, LZ, and X-FG designed and performed the experiments. All authors discussed the results and commented on the manuscript.

## Conflict of Interest

The authors declare that the research was conducted in the absence of any commercial or financial relationships that could be construed as a potential conflict of interest.

## References

[B1] Agrigento P. Albericio F. Chamoin S. Dacquignies I. Koc H. Eberle M. (2014). Facile and mild synthesis of linear and cyclic peptides via thioesters. Org. Lett. 16, 3922–3925. 10.1021/ol501669n 25032897

[B2] Anisfeld S. T. Lansbury P. T. (1990). A convergent approach to the chemical synthesis of asparagine-linked glycopeptides. J. Org. Chem. 55, 5560–5562. 10.1021/jo00308a009

[B3] Aussedat B. Fasching B. Johnston E. Sane N. Nagorny P. Danishefsky S. J. (2012). Total synthesis of the α-subunit of human glycoprotein hormones: toward fully synthetic homogeneous human follicle-stimulating hormone. J. Am. Chem. Soc. 134, 3532–3541. 10.1021/ja2111459 22280541 PMC3288947

[B4] Bhat A. H. Maity S. Giri K. Ambatipudi K. (2019). Protein glycosylation: sweet or bitter for bacterial pathogens? Crit. Rev. Microbiol. 45, 82–102. 10.1080/1040841x.2018.1547681 30632429

[B5] Carubbi F. Alunno A. Gerli R. Giacomelli R. (2019). Post-translational modifications of proteins: novel insights in the autoimmune response in rheumatoid arthritis. Cells 8:657. 10.3390/cells8070657 31261953 PMC6678491

[B6] Chai H. Le Mai Hoang K. Vu M. D. Pasunooti K. Liu C.-F. Liu X.-W. (2016). N-linked glycosyl auxiliary-mediated native chemical ligation on aspartic acid: application towards N-glycopeptide synthesis. Angew. Chem. Int. Ed. 55, 10363–10367. 10.1002/anie.201605597 27444333

[B7] Chen R. Tolbert T. J. (2010). Study of on-resin convergent synthesis of N-linked glycopeptides containing a large high mannose N-linked oligosaccharide. J. Am. Chem. Soc. 132, 3211–3216. 10.1021/ja9104073 20158247

[B8] Chisholm T. S. Kulkarni S. S. Hossain K. R. Cornelius F. Clarke R. J. Payne R. J. (2020). Peptide ligation at high dilution via reductive diselenide-selenoester ligation. J. Am. Chem. Soc. 142, 1090–1100. 10.1021/jacs.9b12558 31840988

[B9] Clerc F. Reiding K. R. Jansen B. C. Kammeijer G. S. Bondt A. Wuhrer M. (2016). Human plasma protein N-glycosylation. Glycoconj. J. 33, 309–343. 10.1007/s10719-015-9626-2 26555091 PMC4891372

[B10] Coffman R. L. Seymour B. W. Hudak S. Jackson J. Rennick D. (1989). Antibody to interleukin-5 inhibits helminth-induced eosinophilia in mice. Science 245, 308–310. 10.1126/science.2787531 2787531

[B11] Cohen-Anisfeld S. T. Lansbury P. T. (1993). A practical, convergent method for glycopeptide synthesis. J. Am. Chem. Soc. 115, 10531–10537. 10.1021/ja00076a010

[B12] Conroy T. Jolliffe K. A. Payne R. J. (2010). Synthesis of N-linked glycopeptides via solid-phase aspartylation. Org. Biomol. Chem. 8, 3723–3733. 10.1039/c003673k 20567757

[B13] Dawson P. E. Muir T. W. Clark-Lewis I. Kent S. B. H. (1994). Synthesis of proteins by native chemical ligation. Science 266, 776–779. 10.1126/science.7973629 7973629

[B14] Du J.-J. Gao X.-F. Xin L.-M. Lei Z. Liu Z. Guo J. (2016). Convergent synthesis of N-linked glycopeptides via aminolysis of ω-Asp *p*-nitrophenyl thioesters in solution. Org. Lett. 18, 4828–4831. 10.1021/acs.orglett.6b02288 27619788

[B15] Du J.-J. Xin L.-M. Lei Z. Zou S.-Y. Xu W.-B. Wang C.-W. (2018). Glycopeptide ligation via direct aminolysis of selenoester. Chin. Chem. Lett. 29, 1127–1130. 10.1016/j.cclet.2018.04.016

[B16] Fairbanks A. J. (2019). Chemoenzymatic synthesis of glycoproteins. Curr. Opin. Chem. Biol. 53, 9–15. 10.1016/j.cbpa.2019.05.015 31202888

[B17] Grieco P. A. Jaw J. Y. Claremon D. Nicolaou K. (1981). N-Phenylselenophthalimide. A useful reagent for the facile transformation of (1) carboxylic acids into either selenol esters or amides and (2) alcohols into alkyl phenyl selenides. J Org Chem. 46, 1215–1217. 10.1021/jo00319a037

[B18] Gui Y. Qiu L. Li Y. Li H. Dong S. (2016). Internal activation of peptidyl prolyl thioesters in native chemical ligation. J. Am. Chem. Soc. 138, 4890–4899. 10.1021/jacs.6b01202 26982082

[B19] Hondal R. J. Nilsson B. L. Raines R. T. (2001). Selenocysteine in native chemical ligation and expressed protein ligation. J. Am Chem. Soc. 123, 5140–5141. 10.1021/ja005885t 11457362

[B20] Joseph R. Dyer F. B. Garner P. (2013). Rapid formation of N-glycopeptides via cu(II)-promoted glycosylative ligation. Org. Lett. 15, 732–735. 10.1021/ol302961s 23346888

[B21] Kajihara Y. Yoshihara A. Hirano K. Yamamoto N. (2006). Convenient synthesis of a sialylglycopeptide-thioester having an intact and homogeneous complex-type disialyl-oligosaccharide. Carbohydr. Res. 341, 1333–1340. 10.1016/j.carres.2006.04.037 16701588

[B22] Kaneshiro C. M. Michael K. (2006). A convergent synthesis of N-glycopeptides. Angew. Chem. Int. Ed. 45, 1077–1081. 10.1002/anie.200502687 16402413

[B23] Kemp D. Vellaccio F. Jr, (1975). Rapid intramolecular acyl transfer from phenol to carbinolamine. Progress toward a new class of peptide coupling reagent. Org. Chem. 40, 3003–3004. 10.1021/jp3083858 1177079

[B24] Kent S. B. H. (2009). Total chemical synthesis of proteins. Chem. Soc. Rev. 38, 338–351. 10.1039/b700141j 19169452

[B25] Kunz H. Unverzagt C. (1988). Protecting-group-dependent stability of intersaccharide bonds-synthesis of a fucosyl-chitobiose glycopeptide. Angew. Chem. Int. Ed. 27, 1697–1699. 10.1002/anie.198816971

[B26] Lee C. L. Liu H. Wong C. T. T. Chow H. Y. Li X. (2016). Enabling N-to-C Ser/Thr ligation for convergent protein synthesis via combining chemical ligation approaches. J. Am. Chem. Soc. 138, 10477–10484. 10.1021/jacs.6b04238 27479006

[B27] Li X. Lam H. Y. Zhang Y. Chan C. K. (2010). Salicylaldehyde ester-induced chemoselective peptide ligations: enabling generation of natural peptidic linkages at the serine/threonine sites. Org. Lett. 12, 1724–1727. 10.1021/ol1003109 20232847

[B28] Li Y. Tran A. H. Danishefsky S. J. Tan Z. (2019). Chemical biology of glycoproteins: From chemical synthesis to biological impact. Methods Enzymol. 621, 213–229. 10.1016/bs.mie.2019.02.030 31128780 PMC6595224

[B29] Likhosherstov L. M. Novikova O. S. Derevitskaja V. A. Kochetkov N. K. (1986). A new simple synthesis of amino sugar β-D-glycosylamines. Carbohydr. Res. 146, C1–C5. 10.1016/0008-6215(86)85037-6

[B30] LingáTung C. Clarence T. (2015). Peptide 2-formylthiophenol esters do not proceed through a Ser/Thr ligation pathway, but participate in a peptide aminolysis to enable peptide condensation and cyclization. Org. Biomol. Chem. 13, 6922–6926. 10.1039/C5OB00825E 26013965

[B31] Liu J. Dong S. (2018). Synthetic studies toward human interleukin-5. Chin. Chem. Lett. 29, 1131–1134. 10.1016/j.cclet.2018.05.014

[B32] Mezzato S. Schaffrath M. Unverzagt C. (2005). An orthogonal double-linker resin facilitates the efficient solid-phase synthesis of complex-type N-glycopeptide thioesters suitable for native chemical ligation. Angew. Chem. Int. Ed. 44, 1650–1654. 10.1002/anie.200461125 15693053

[B33] Mitchell N. J. Malins L. R. Liu X. Thompson R. E. Chan B. Radom L.. (2015). Rapid additive-free selenocystine–selenoester peptide ligation. J. Am. Chem. Soc. 137, 14011–14014. 10.1021/jacs.5b07237 26487084

[B34] Nagorny P. Sane N. Fasching B. Aussedat B. Danishefsky S. J. (2012). Probing the frontiers of glycoprotein synthesis: the fully elaborated β-subunit of the human follicle-stimulating hormone. Angew. Chem. Int. Ed. 51, 975–979. 10.1002/anie.201107482 22162182 PMC3285374

[B35] Offer J. Quibell M. Johnson T. (1996). On-resin solid-phase synthesis of asparagine N-linked glycopeptides: use of N-(2-acetoxy-4-methoxybenzyl) (AcHmb) aspartyl amide-bond protection to prevent unwanted aspartimide formation. J. Chem. Soc. Perkin Trans. 1, 175–182. 10.1039/P19960000175

[B36] Okamoto R. Izumi M. Kajihara Y. (2014a). Decoration of proteins with sugar chains: recent advances in glycoprotein synthesis. Curr. Opin. Chem. Biol. 22, 92–99. 10.1016/j.cbpa.2014.09.029 25291353

[B37] Okamoto R. Mandal K. Ling M. Luster A. D. Kajihara Y. Kent S. B. H. (2014b). Total chemical synthesis and biological activities of glycosylated and non-glycosylated forms of the chemokines CCL1 and Ser-CCL1. Angew. Chem. Int. Ed. 53, 5188–5193. 10.1002/anie.201310574 24644239

[B38] Oliveira-Ferrer L. Legler K. Milde-Langosch K. (2017). Role of protein glycosylation in cancer metastasis. Semin. Cancer Biol. 44, 141–152. 10.1016/j.semcancer.2017.03.002 28315783

[B39] Park S. S. Park J. Ko J. Chen L. Meriage D. Crouse-Zeineddini J.. (2009). Biochemical assessment of erythropoietin products from Asia versus US Epoetin alfa manufactured by Amgen. J. Pharm. Sci. 98, 1688–1699. 10.1002/jps.21546 18781649

[B40] Payne R. J. Ficht S. Greenberg W. A. Wong C. H. (2008). Cysteine-free peptide and glycopeptide ligation by direct aminolysis. Angew. Chem. Int. Ed. 47, 4411–4415. 10.1002/anie.200705298 18442150

[B41] Payne R. J. Wong C.-H. (2010). Advances in chemical ligation strategies for the synthesis of glycopeptides and glycoproteins. Chem. Commun. 46, 21–43. 10.1039/B913845E 20024291

[B42] Piontek C. Ring P. Harjes O. Heinlein C. Mezzato S. Lombana N.. (2009a). Semisynthesis of a homogeneous glycoprotein enzyme: ribonuclease C: Part 1. Angew. Chem. Int. Ed. 48, 1936–1940. 10.1002/anie.200804734 19173366

[B43] Piontek C. Varón Silva D. Heinlein C. Pöhner C. Mezzato S. Ring P.. (2009b). Semisynthesis of a homogeneous glycoprotein enzyme: ribonuclease C: Part 2. Angew. Chem. Int. Ed. 48, 1941–1945. 10.1002/anie.200804735 19180621

[B44] Raj M. Wu H. Blosser S. L. Vittoria M. A. Arora P. S. (2015). Aldehyde capture ligation for synthesis of native peptide bonds. J. Am. Chem. Soc. 137, 6932–6940. 10.1021/jacs.5b03538 25966041

[B45] Reif A. Siebenhaar S. Tröster A. Schmälzlein M. Lechner C. Velisetty P.. (2014). Semisynthesis of biologically active glycoforms of the human cytokine interleukin 6. Angew. Chem. Int. Ed. 53, 12125–12131. 10.1002/anie.201407160 25243720

[B46] Sakamoto I. Tezuka K. Fukae K. Ishii K. Taduru K. Maeda M.. (2012). Chemical synthesis of homogeneous human glycosyl-interferon-β that exhibits potent antitumor activity *in vivo*. J. Am. Chem. Soc. 134, 5428–5431. 10.1021/ja2109079 22404596

[B47] Sayers J. Karpati P. M. Mitchell N. J. Goldys A. M. Kwong S. M. Firth N.. (2018a). Construction of challenging proline–proline junctions via diselenide–selenoester ligation chemistry. J. Am. Chem. Soc. 140, 13327–13334. 10.1021/jacs.8b07877 30239198

[B48] Sayers J. Payne R. J. Winssinger N. (2018b). Peptide nucleic acid-templated selenocystine-selenoester ligation enables rapid miRNA detection. Chem. Sci. 9, 896–903. 10.1039/C7SC02736B 29629156 PMC5873163

[B49] Schöwe M. J. Keiper O. Unverzagt C. Wittmann V. (2019). A Tripeptide approach to the solid-phase synthesis of peptide thioacids and N-glycopeptides. Chem. Eur. J. 25, 15759–15764. 10.1002/chem.201904688 31628819 PMC6916195

[B50] Seko A. Koketsu M. Nishizono M. Enoki Y. Ibrahim H. R. Juneja L. R. (1997). Occurrence of a sialylglycopeptide and free sialylglycans in hen's egg yolk. Biochim. Biophys. Acta Gen. Subj. 1335, 23–32. 10.1016/S0304-4165(96)00118-3 9133639

[B51] Sun B. Bao W. Tian X. Li M. Liu H. Dong J.. (2014). A simplified procedure for gram-scale production of sialylglycopeptide (SGP) from egg yolks and subsequent semi-synthesis of Man3GlcNAc oxazoline. Carbohydr. Res. 396, 62–69. 10.1016/j.carres.2014.07.013 25124522

[B52] Takei T. Andoh T. Takao T. Hojo H. (2017). One-pot four-segment ligation using seleno- and thioesters: synthesis of superoxide dismutase. Angew. Chem. Int. Ed. 56, 15708–15711. 10.1002/anie.201709418 29048715

[B53] Temperini A. Piazzolla F. Minuti L. Curini M. Siciliano C. (2017). General, mild, and metal-free synthesis of phenyl selenoesters from anhydrides and their use in peptide synthesis. J. Org. Chem. 82, 4588–4603. 10.1021/acs.joc.7b00173 28414443

[B54] Ullmann V. Rädisch M. Boos I. Freund J. Pöhner C. Schwarzinger S.. (2012). Convergent solid-phase synthesis of N-glycopeptides facilitated by pseudoprolines at consensus-sequence Ser/Thr residues. Angew. Chem. Int. Ed. 51, 11566–11570. 10.1002/anie.201204272 22945377

[B55] Varki A. (2017). Biological roles of glycans. Glycobiology 27, 3–49. 10.1093/glycob/cww086 27558841 PMC5884436

[B56] Vetter D. Tumelty D. Singh S. K. Gallop M. A. (1995). A versatile solid-phase synthesis of N-linked glycopeptides. Angew. Chem. Int. Ed. 34, 60–63. 10.1002/anie.199500601

[B57] Walsh G. Jefferis R. (2006). Post-translational modifications in the context of therapeutic proteins. Nat. Biotechnol. 24, 1241–1252. 10.1038/nbt1252 17033665

[B58] Wan Q. Chen J. Yuan Y. Danishefsky S. J. (2008). Oxo-ester mediated native chemical ligation: concept and applications. J. Am Chem. Soc. 130, 15814–15816. 10.1021/ja804993y 18855357 PMC2645925

[B59] Wang L.-X. Amin M. N. (2014). Chemical and chemoenzymatic synthesis of glycoproteins for deciphering functions. Chem. Biol. 21, 51–66. 10.1016/j.chembiol.2014.01.001 24439206 PMC3923302

[B60] Wang P. Aussedat B. Vohra Y. Danishefsky S. J. (2012). An advance in the chemical synthesis of homogeneous N-linked glycopolypeptides by convergent aspartylation. Angew. Chem. Int. Ed. 51, 11571–11575. 10.1002/anie.201205038 23011954 PMC3500778

[B61] Wang P. Dong S. Shieh J.-H. Peguero E. Hendrickson R. Moore M. A. S.. (2013). Erythropoietin derived by chemical synthesis. Science 342, 1357–1360. 10.1126/science.1245095 24337294 PMC4080428

[B62] Wang P. Li X. Zhu J. Chen J. Yuan Y. Wu X.. (2011). Encouraging progress in the ω-aspartylation of complex oligosaccharides as a general route to β-N-linked glycopolypeptides. J. Am. Chem. Soc. 133, 1597–1602. 10.1021/ja110115a 21207981 PMC3033444

[B63] Wang X. Corcilius L. Premdjee B. Payne R. J. (2020). Synthesis and utility of β-selenophenylalanine and β-selenoleucine in diselenide–selenoester ligation. J Org. Chem. 85, 1567–1578. 10.1021/acs.joc.9b02665 31840993

[B64] Wilson R. M. Dong S. Wang P. Danishefsky S. J. (2013). The winding pathway to erythropoietin along the chemistry–biology frontier: a success at last. Angew. Chem. Int. Ed. 52, 7646–7665. 10.1002/anie.201301666 23775885 PMC4729195

[B65] Yamamoto N. Tanabe Y. Okamoto R. Dawson P. E. Kajihara Y. (2008). Chemical synthesis of a glycoprotein having an intact human complex-type sialyloligosaccharide under the boc and fmoc synthetic strategies. J. Am. Chem. Soc. 130, 501–510. 10.1021/ja072543f 18085777

[B66] Yin X.-G. Gao X.-F. Du J.-J. Zhang X.-K. Chen X.-Z. Wang J.. (2016). Preparation of protein conjugates via homobifunctional diselenoester cross-linker. Org. Lett. 18, 5796–5799. 10.1021/acs.orglett.6b02568 27934486

[B67] Zou Y. Hu J. Jie J. Lai J. Li M. Liu Z.. (2020). Comprehensive analysis of human IgG Fc N-glycopeptides and construction of a screening model for colorectal cancer. J. Proteomics 213:103616. 10.1016/j.jprot.2019.103616 31846768

