# Supplementary material for: Peptidyl ω-Asp Selenoesters Enable Efficient Synthesis of N-Linked Glycopeptides
Source: Front Chem. 2020 May 5;8:396. doi: 10.3389/fchem.2020.00396 (PMC7232547; doi:10.3389/fchem.2020.00396)
Supplement: Supplementary file 1 [file Data_Sheet_1.PDF]

Supplementary Material

Peptidyl *ω*-Asp Selenoesters Enable Efficient Synthesis of *N*-linked Glycopeptides

Jing-Jing Du[1†], Lian Zhang[1†], Xiao-Fei Gao[2], Hui Sun[3], and Jun Guo[1*]

[1]Key Laboratory of Pesticide and Chemical Biology of Ministry of Education, Hubei International Scientific and Technological Cooperation Base of Pesticide and Green Synthesis, International Joint Research Center for Intelligent Biosensing Technology and Health, College of chemistry, Central China Normal University, Wuhan, Hubei 430079, China
[2]Jiangxi Key Laboratory for Mass Spectrometry and Instrumentation, East China University of Technology, Nanchang, Jiangxi 330013, China
[3]Hubei Key Laboratory of Cell Homeostasis, College of Life Sciences, Hubei Province key Laboratory of Allergy and Immunology, and Key laboratory of Combinatorial Biosynthesis and Drug Discovery, Ministry of Education, Wuhan University, Wuhan, Hubei 430071, China
†J.-J.D. and L.Z. contributed equally to this work

***Correspondence:** jguo@mail.ccnu.edu.cn

Table of contents

1. General information 2

2. Characterization of compounds 3

3. Analysis of the process of Fmoc-Gly-ester-mediated glycosylation 4

4. Analysis of the process of peptidyl selenoester-mediated glycosylation 6

5. Analysis of the process of peptidyl selenoester-mediated glycosylation containing the complex undecasaccharide 13

6. HPLC and ESI-MS spectra of peptidyl selenoesters 17

7. HPLC and ESI-MS spectra of *N*-linked glycopeptides 23

8. ^{1}H NMR and ^{13}C NMR spectra 30

9. Supplementary references 32

Supplementary Material

1. General Information

^{1}H-NMR spectra were recorded on a Varian Mercury Plus 400 MHz or 600 MHz instrument. ^{13}C-NMR spectra were recorded on this same instrument at 100 MHz or 150 MHz. Chemical shifts (d) are expressed in ppm downfield from TMS as internal standard. The letters s, brs, t, dd and m are used to indicate singlet, broad singlet, triplet, double of doublets, and multiplet, respectively. Electrospray ionization mass (ESI-MS) was performed on TSQ Quantum Access MAX (ThermoFisher Scientific). Analytical HPLC (Agilent, 1100 series HPLC system) analysis was carried out on a C18 reversed-phase analytical column (Agilent, 250 × 4.6 mm, particle size 5 μm) at 35℃, involved a mobile phase A (water + 0.1% TFA) and B (acetonitrile + 0.1% TFA) at a flow rate of 1.0 mL/min. Semi-preparative HPLC analysis was performed on C18 reversed-phase column (Agilent 250 × 9.4 mm, particle size 5 μm) at 35℃, involving a mobile phase A and B at a flow rate of 4.0 mL/min. All solvents and chemicals were obtained from standard commercial suppliers and without any further purification.

2-Chlorotrityl chloride resin, all amino acids and beznotriazole-1-yl-oxy-tris-pyrrolidino-phosphonium hexafluorophosphate (PyBOP), 1-(3-dimethylaminopropyl)-3-ethylcarbodiimide hydrochloride (EDCI), *N*,*N*-diisopropylethylamine (DIPEA), trifluoroacetic acid (TFA) and triisopropylsilane (TIPS) were obtained from InnoChem Science & Technology Co., Ltd. *N*,*N*-dimethylformamide (DMF), dimethyl sulfoxide (DMSO), DCM and THF were purchased from Aldrich and dried before use. The glycosylamines (**1a-1c**), FmocGly-COOPh, FmocGly-COSPh, FmocGly-COS-*p*-PhNO$_2$, FmocGly-COSePh and FmocGly-COSe-*o*-CHO, FmocGly-COSe-*p*-CHO were synthesized and characterized by the reported procedure[1,2].

General procedure for peptide synthesis

2-Chlorotrityl chloride resin (1.06 mmol/g) was swollen in dry DCM for 30 min, and washed with DCM (3 × 3 mL) and DMF (3 × 3 mL). Followed by a solution of Fmoc-AA-OH and DIPEA in DMF was added to the resin bubbling with N_2 and shaken at room temperature for 10 h. The resin was washed with DMF (3 × 3 mL), DCM (3 × 3 mL) and DMF (3 × 3 mL) and subsequently submitted to iterative peptide assembly. After deprotection with 20% piperidine/DMF (3 mL, 3 × 5 min), washed with DMF (3 × 3 mL), DCM (3 × 3 mL) and DMF (3 × 3 mL). Next, a solution of PyBOP and DIPEA in DMF were used for Fmoc/Boc-AA-OH coupling to resin, after shaking at room temperature for 2 h, the resin was washed with DMF (3 × 3 mL), DCM (3 × 3 mL) and DMF (3 × 3 mL).

General procedure for the synthesis of peptidyl selenoester

Peptidyl selenoester was synthesized manually on 2-chlorotrityl chloride resin as the general procedure, followed by removal of allyl ester under the catalytic amounts of $Pd(PPh_3)_4$ (0.2 equiv) in the presence of $PhSiH_3$ (10.0 equiv) were dissolved in dry DCM bubbling with N_2 and shaken for 30 min. Next, the resin was washed with dry DCM and the process was repeated once. After 1 h, the resin was washed with DMF (3 × 3 mL), DCM (3 × 3 mL) and DMF (3 × 3 mL). In addition, on-resin selenoesterification for the synthesis of peptidyl selenoester was conducted by adding diphenyl diselenide (DPDS, 2.0 equiv) in a dried round bottom flask, thoroughly flushed with the argon. Then, dry DMF and tributylphosphine (5.0 equiv) were added. The resulting suspension was bubbled for 4 h. The resin was then filtered and washed with DCM/DMF. A mixture of TFA and DCM (1:100 v/v) was added. After 1 h, the resin was washed with CH_2Cl_2 (2 × 2 mL). The combined filtrates were

concentrated and the crude product was triturated with cold diethyl ether to give a white suspension, which was centrifuged and the ether subsequently decanted to obtain crude peptidyl selenoester. The crude peptidyl selenoester was purified by semi-preparative HPLC and lyophilised.

General procedure for the synthesis of *N*-linked glycopeptide with monosaccharide or chitobiose

The monosaccharide (**1a**) or chitobiose (**1b**) (10 μmol) was dissolved in DMSO (1 mL) and treated with DIPEA (10 μmol). Then, the purified peptidyl selenoesters (**4b-9b**) (5 μmol) were added, the resulting mixture was stirred at room temperature for 2 h. The reaction processes were monitored by HPLC. Upon completion of the coupling, crude products were purified by semi-preparative HPLC equipped with a C18 column (Agilent 250 × 9.2 mm, particle size 5 μm), involving mobile phases of 0.1% TFA (v/v) in water (solvent A)/0.1% TFA in acetonitrile (solvent B) at a flow rate of 4.0 mL/min, then lyophilized. The *N*-linked glycopeptides (**4c-7c, 5d, 8d and 9d**) were obtained as white powders with 69 to 84% yield.

General procedure for the synthesis of complex *N*-linked glycopeptide with undecasaccharide

The undecasaccharide (**1c**, 1.5 μmol) was dissolved in DMSO (0.5 mL) and 4 Å MS (2.0 mg), the peptidyl selenoester (1 μmol) and DIPEA (2 μmol) were added, the resulting mixture was stirred at room temperature for 6 h. The reaction processes were monitored by HPLC. Upon completion of the coupling, crude products were purified by semi-preparative HPLC equipped with a C18 column (Agilent 250 × 9.2 mm, particle size 5 μm), involving mobile phases of 0.1% TFA (v/v) in water (solvent A)/0.1% TFA in acetonitrile (solvent B) at a flow rate of 4.0 mL/min, then lyophilized. The complex *N*-linked Glycopeptides (**10e-12e**) were obtained as white powders with 59 to 65% yield.

2. Characterization of Compounds

Seleno-phenyl ester (2d)

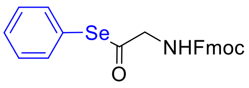

2d

86% isolated yield. **^{1}H NMR** (600 MHz, $CDCl_3$) δ (ppm) = 7.78 (d, *J* = 7.5 Hz, 2 H), 7.64 (d, *J* = 7.5 Hz, 2 H), 7.50 (d, *J* = 6.7 Hz, 2 H), 7.44−7.38 (m, 5 H), 7.33 (t, *J* = 7.1 Hz, 2 H), 5.52 (br. s., 1 H), 4.51 (d, *J* = 6.7 Hz, 2 H), 4.30−4.25 (m, 1 H), 4.14 (d, *J* = 5.9 Hz, 2 H). **^{13}C NMR** (100 MHz, $CDCl_3$) δ = 200.0, 156.0, 143.4, 141.1, 135.8, 129.3, 129.0, 127.6, 126.9, 124.8, 119.9, 77.3, 76.7, 67.4, 53.6, 47.2. ESI-MS calcd for **2d** $C_{23}H_{18}N_2O_5S$ $[M + H]^+$ m/z = 438.06, found: 438.02.

Seleno-benzaldehyde ester (2e)

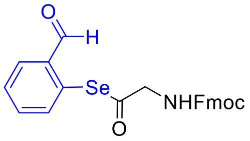

2e

80% isolated yield. **^{1}H NMR** (400 MHz, $CDCl_3$) δ (ppm) = 10.08 (s, 1 H), 8.09−7.93 (m, 1 H), 7.74 (d, *J* = 7.3 Hz, 2 H), 7.64−7.48 (m, 5 H), 7.40−7.25 (m, 4 H), 5.90 (br. s., 1 H), 4.52 (d, *J* = 6.7 Hz, 2 H), 4.25 (t, *J* = 6.4 Hz, 1 H), 4.10 (d, *J* = 6.1 Hz, 2 H); **^{13}C NMR** (100 MHz, $CDCl_3$) δ = 199.4, 192.4, 156.1, 143.3, 141.0, 137.7, 136.6, 134.1, 129.7, 129.4, 129.2, 127.5, 126.9, 124.8, 119.8, 77.3, 76.7, 67.3, 53.5, 47.1. ESI-MS calcd for **2e** $C_{23}H_{18}N_2O_5S$ $[M + H]^+$ m/z = 466.05, found: 466.09.

Seleno-benzaldehyde ester (2f)

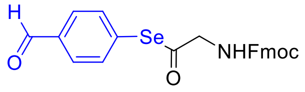

2f

83% isolated yield. ^{1}**H NMR** (400 MHz, $CDCl_3$) δ (ppm) = 10.02 (s, 1 H), 7.87 (d, *J* = 8.5 Hz, 2 H), 7.77 (d, *J* = 7.3 Hz, 2 H), 7.68–7.59 (m, 4 H), 7.43–7.37 (m, 2 H), 7.35–7.30 (m, 2 H), 5.42 (br. s., 1 H), 4.56 (d, *J* = 6.7 Hz, 2 H), 4.32–4.23 (m, 1 H), 4.17 (d, *J* = 5.5 Hz, 2 H); ^{13}**C NMR** (100 MHz, $CDCl_3$) δ = 191.3, 185.2, 156.1, 143.3, 141.1, 136.0, 130.6, 130.1, 129.8, 127.6, 126.9, 124.8, 119.9, 77.3, 76.7, 67.4, 53.6, 47.1. ESI-MS calcd for **2f** $C_{23}H_{18}N_2O_5S$ $[M + H]^+$ m/z = 466.05, found: 466.02.

***N*-linked glycopeptide (4c)[1,3]**

N-linked glycopeptide **4c** as a model of glycosylation reaction, ^{1}**H NMR** (600 MHz, DMSO-d_6) δ (ppm) = 8.53 (d, J = 8.1 Hz, 2H), 8.32 (d, J = 6.6 Hz, 2H), 8.19 (d, J = 9.1 Hz, 2H), 7.99 (t, J = 10.3 Hz, 3H), 7.78 (d, J = 10.0 Hz, 2H), 7.36–7.16 (m, 10H), 7.15–7.06 (m, 3H), 7.02–6.95 (m, 2H), 6.66 (s, 2H), 5.32 (dd, J = 5.5, 4.2 Hz, 3H), 4.81 (t, J = 9.3 Hz, 1H) (β-anomer), 4.55 (q, J = 6.5 Hz, 3H), 4.37 (q, J = 7.8 Hz, 2H), 4.22 (t, J = 7.7 Hz, 1H), 4.19–4.12 (m, 2H), 4.11–4.07 (m, 1H), 3.92 (q, J = 7.9 Hz, 3H), 3.64 (s, 1H), 3.57–3.49 (m, 3H), 3.08 (s, 3H), 2.98 (d, J = 10.3 Hz, 2H), 2.75–2.60 (m, 5H), 2.39–2.29 (m, 3H), 2.04–1.95 (m, 4H), 1.78 (s, 1H), 1.70 (q, J = 6.8 Hz, 3H), 1.51–1.42 (m, 5H), 1.36 (d, J = 14.9 Hz, 4H), 1.30–1.21 (m, 11H), 1.15 (dd, J = 21.8, 6.6 Hz, 4H), 1.04 (d, J = 6.2 Hz, 1H), 0.91–0.81 (m, 5H), 0.81–0.75 (m, 3H).

3. Analysis of the Process of Fmoc-Gly-ester-mediated Glycosylation[1]

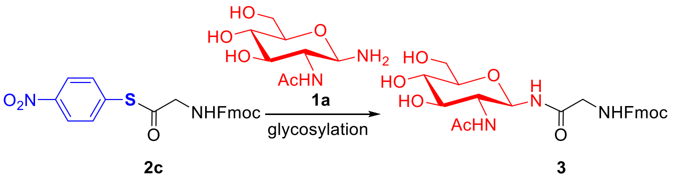

(a)

(b)

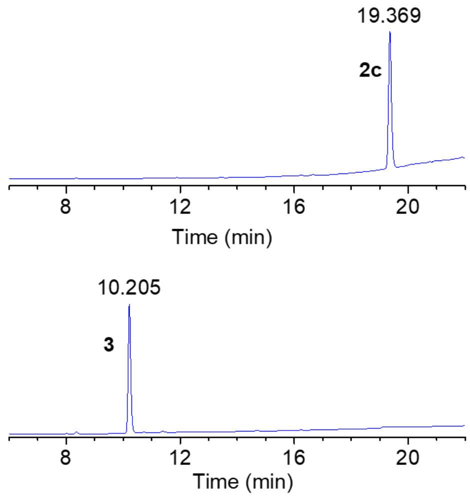

Figure S1. HPLC spectra of the starting material and the crude reaction mixture after 2 h of incubation; the product peak is labeled (gradient: 30% to 90% CH_3CN over 22 min, λ = 254 nm).

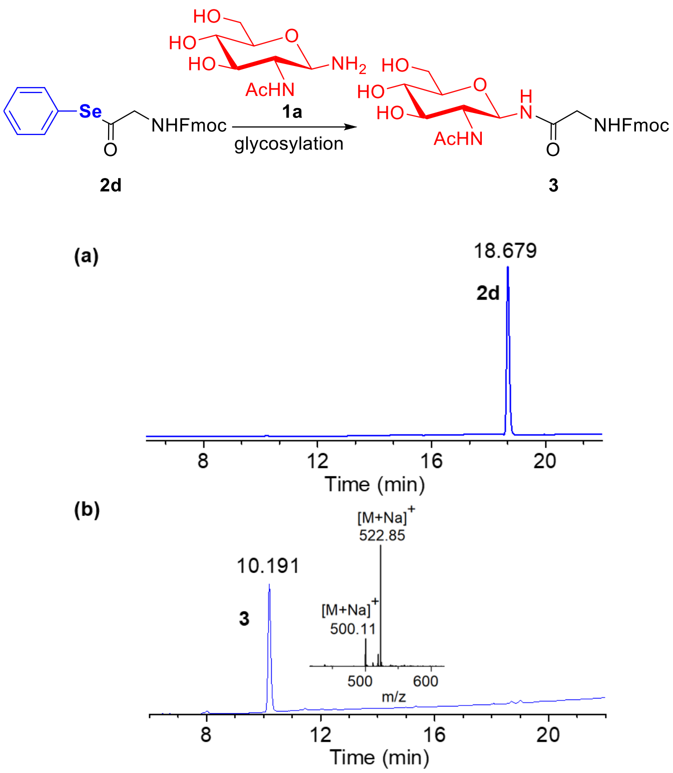

Figure S2. HPLC spectra of the starting material and the crude reaction mixture after 2 h of incubation; the product peak is labeled (gradient: 30% to 90% CH_3CN over 22 min, λ = 254 nm).

4. Analysis of the Process of Peptidyl Selenoester-mediated Glycosylation[1]

4.1 Synthesis of glycopeptide 4c[3]

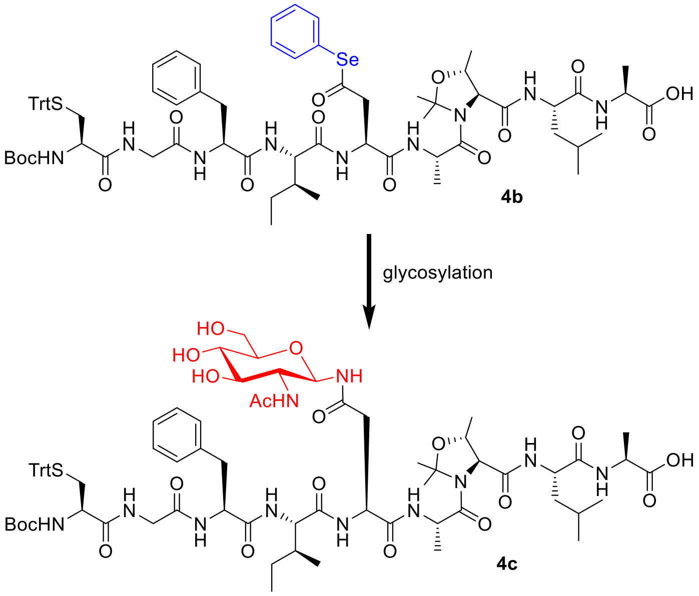

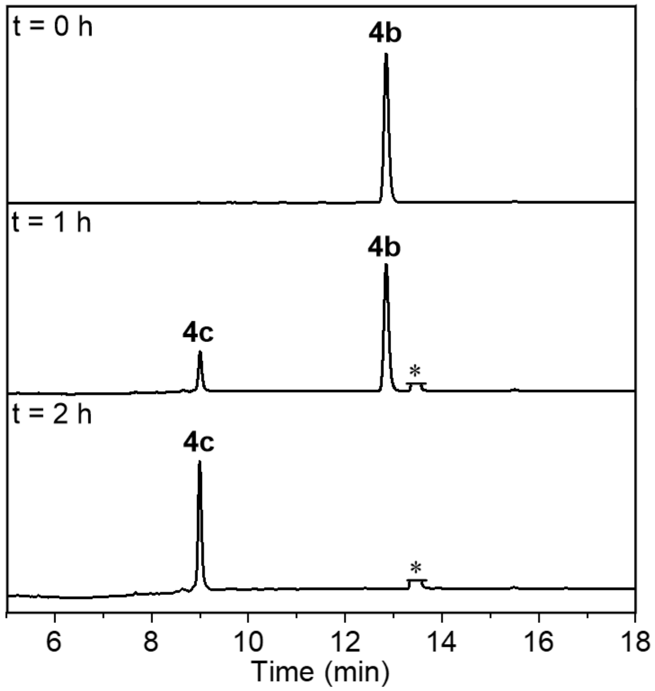

Figure S3. Analytical HPLC profiles of the ligation reaction (gradient: 80% to 100% CH_3CN over 18 min, λ = 254 nm): *t* = 0 h, *t* = 1 h, *t* = 2 h (* is the DPDS) (the hydrolyzed **4b** segment was not detected during the reaction).

4.2 Synthesis of glycopeptide 5c

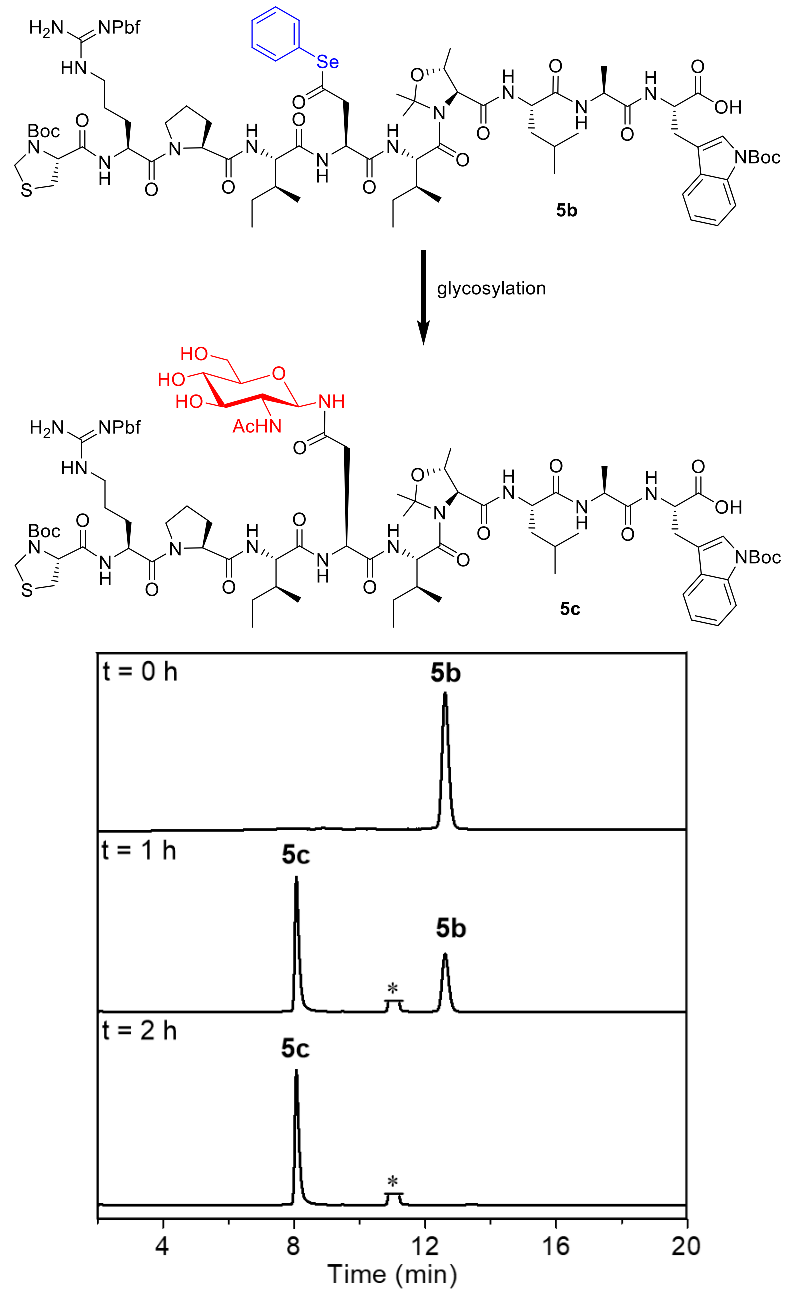

Figure S4. Analytical HPLC profiles of the ligation reaction (gradient: 90% CH_3CN over 20 min, λ = 254 nm): t = 0 h, t = 1 h, t = 2 h (* is the DPDS) (the hydrolyzed **5b** segment was not detected during the reaction).

4.3 Synthesis of glycopeptide 6c

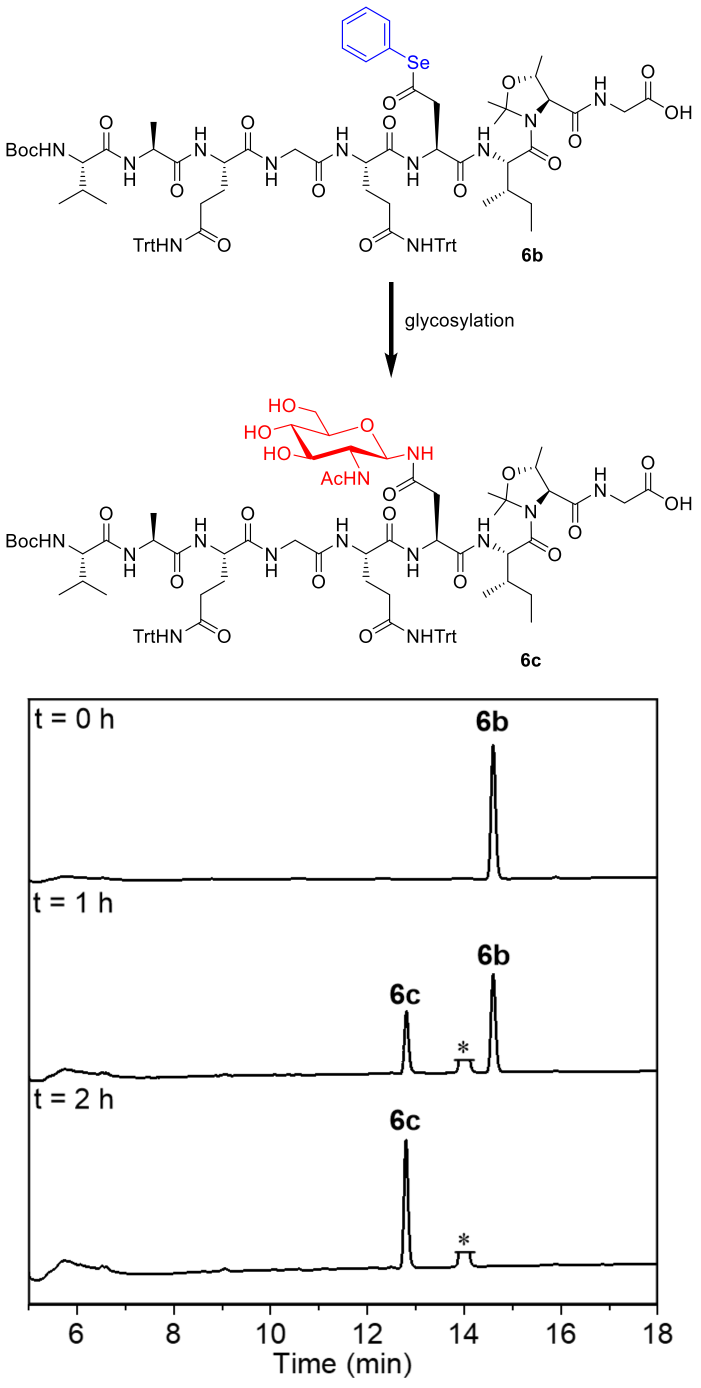

Figure S5. Analytical HPLC profiles of the ligation reaction (gradient: 75% to 100% CH_3CN over 18 min, λ = 254 nm): t = 0 h, t = 1 h, t = 2 h (* is the DPDS) (the hydrolyzed **6b** segment was not detected during the reaction).

4.4 Synthesis of glycopeptide 7c

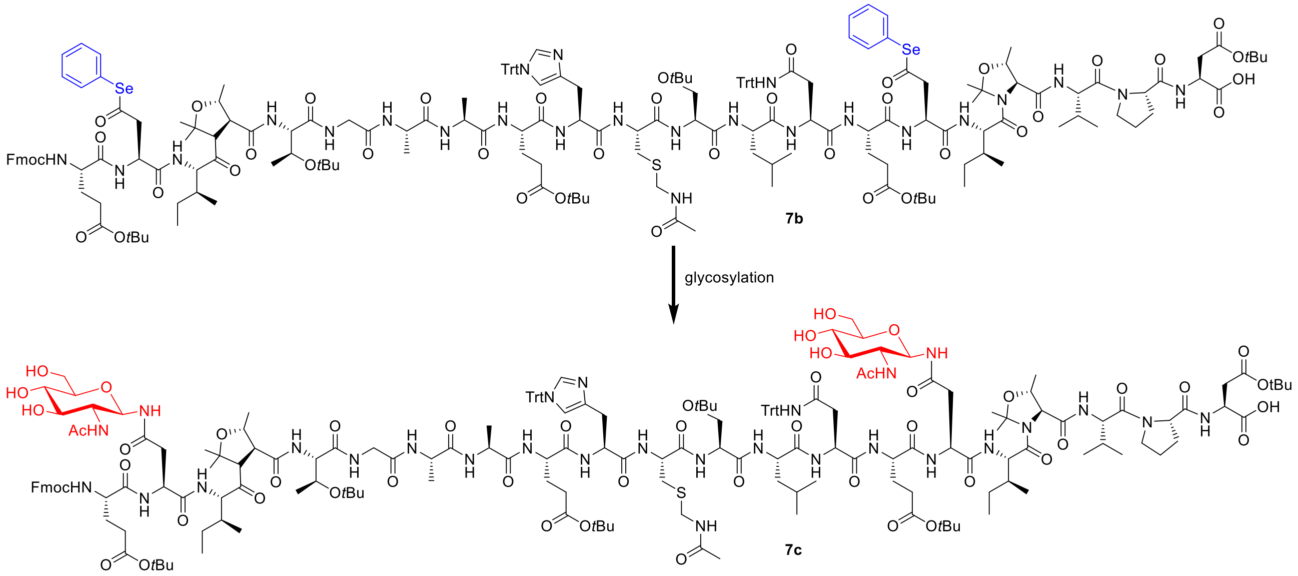

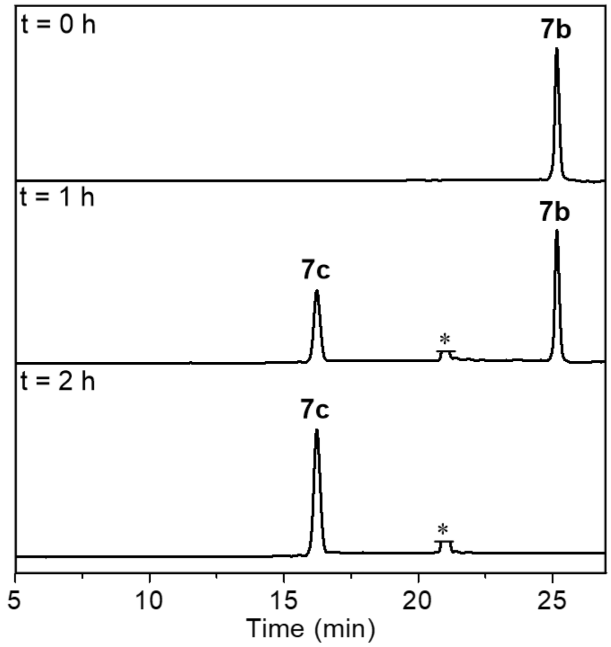

Figure S6. Analytical HPLC profiles of the ligation reaction (gradient: 50% to 100% CH_3CN over 27 min, λ = 254 nm): *t* = 0 h, *t* = 1 h, *t* = 2 h (* is the DPDS) (the hydrolyzed **7b** segment was not detected during the reaction).

4.5 Synthesis of glycopeptide 5d

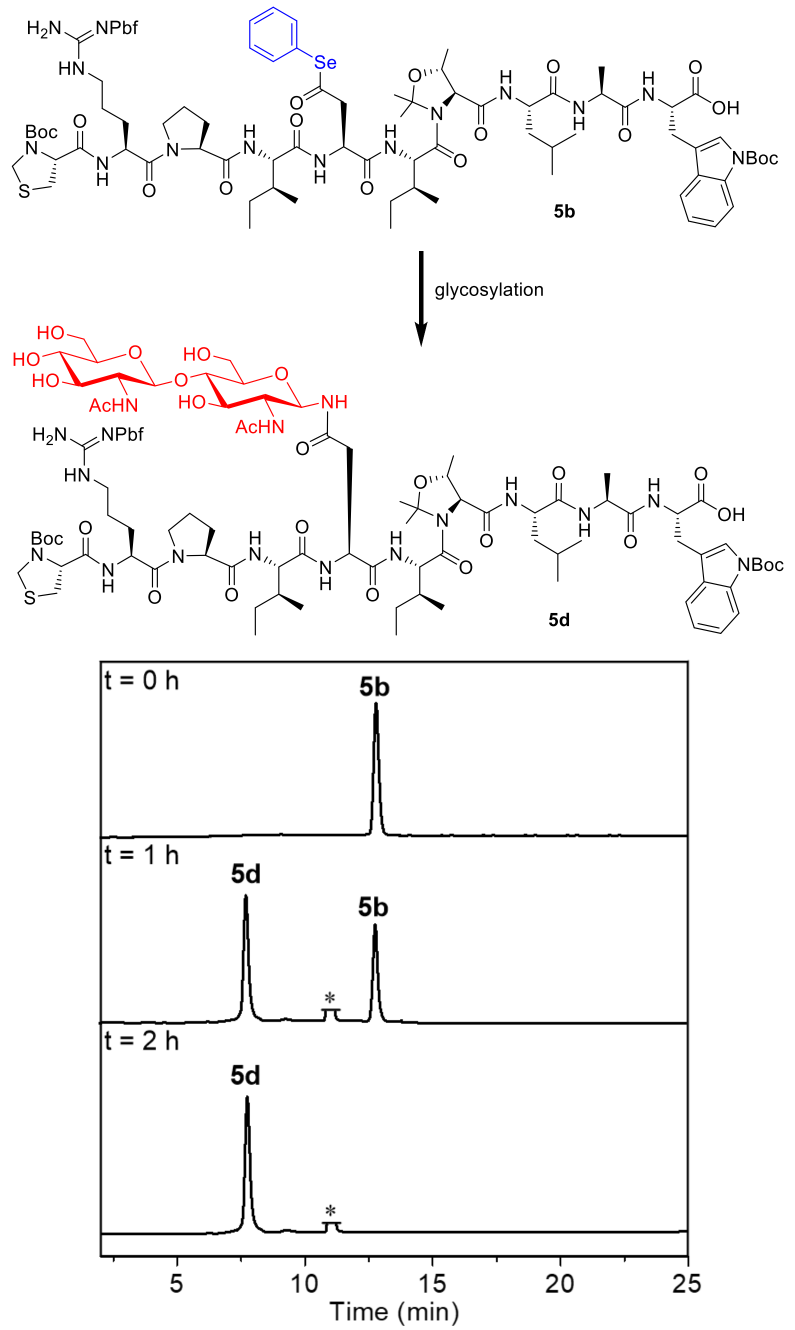

Figure S7. Analytical HPLC profiles of the ligation reaction (gradient: 90% CH_3CN over 25 min, λ = 254 nm): t = 0 h, t = 1 h, t = 2 h (* is the DPDS) (the hydrolyzed **5b** segment was not detected during the reaction).

4.6 Synthesis of glycopeptide 8d

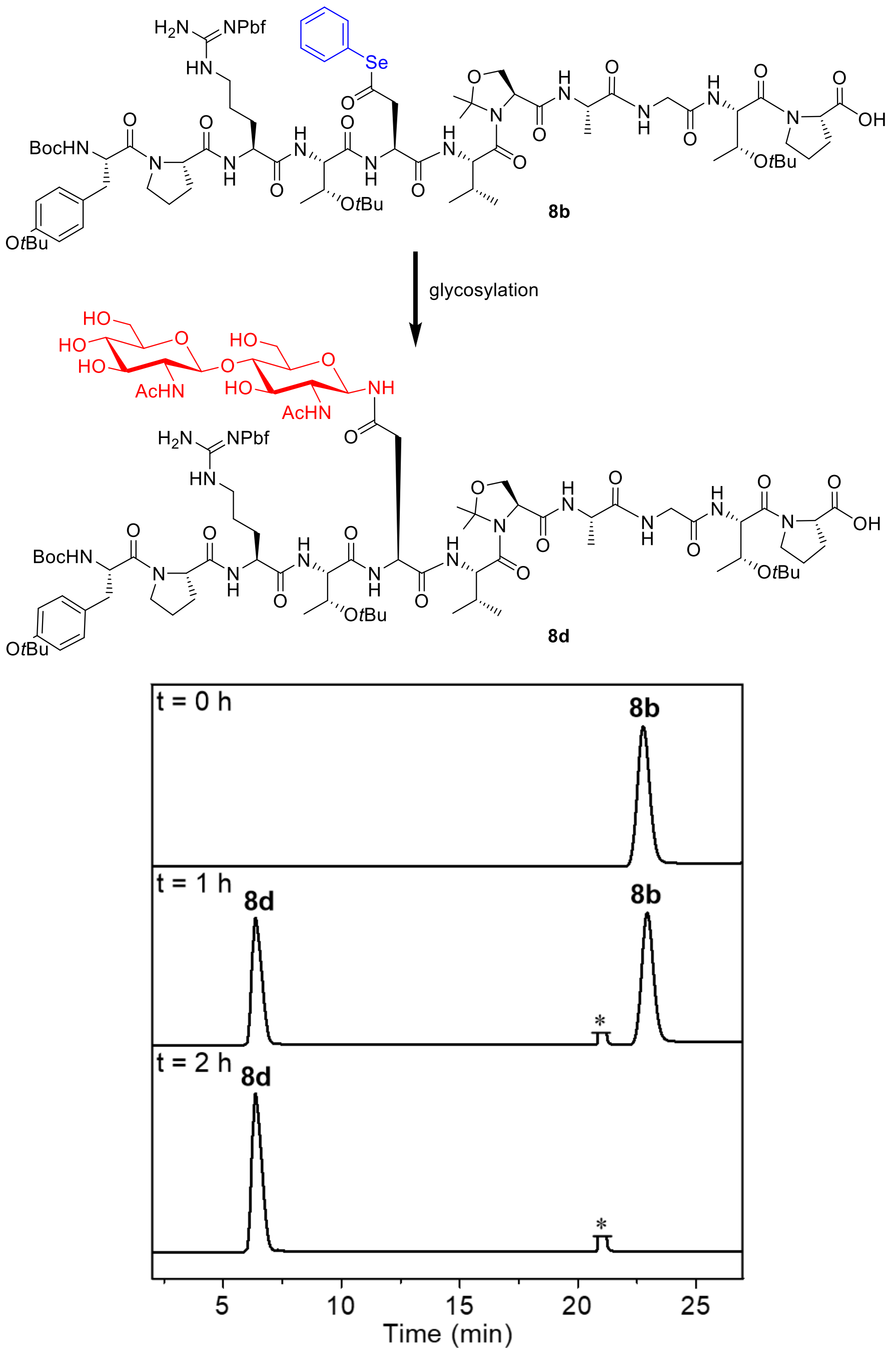

Figure S8. Analytical HPLC profiles of the ligation reaction (gradient: 50% to 100% CH_3CN over 27 min, λ = 254 nm): t = 0 h, t = 1 h, t = 2 h (* is the DPDS) (the hydrolyzed **8b** segment was not detected during the reaction).

4.7 Synthesis of glycopeptide 9d

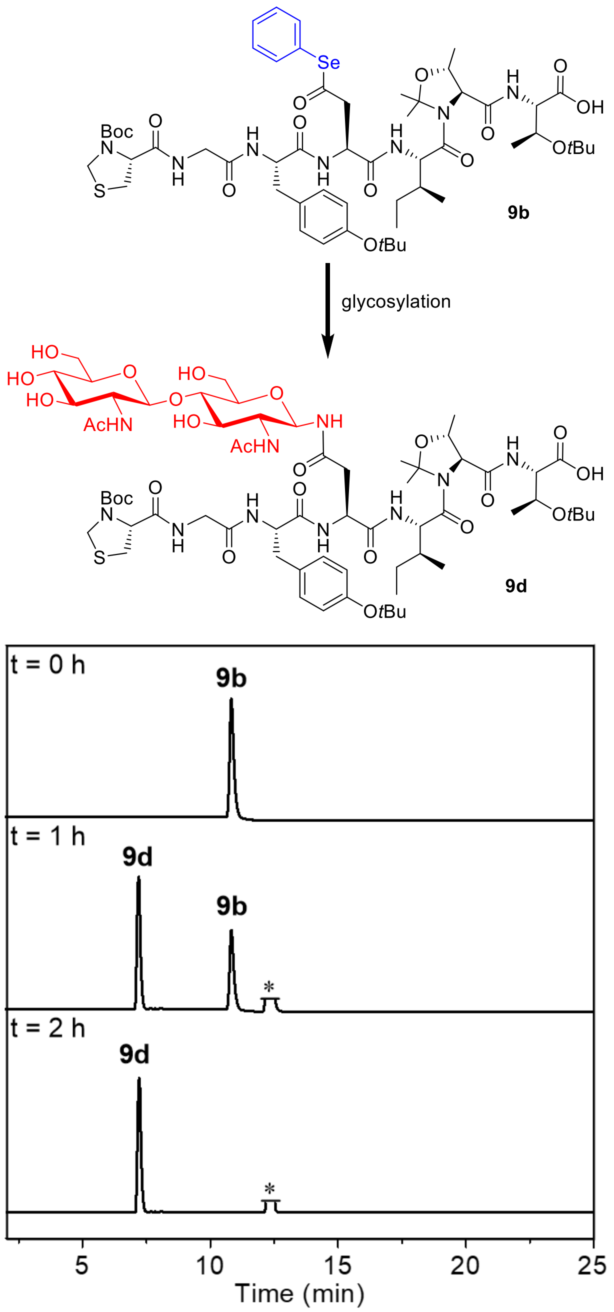

Figure S9. Analytical HPLC profiles of the ligation reaction (gradient: 85% to 100% CH_3CN over 25 min, λ = 254 nm): *t* = 0 h, *t* = 1 h, *t* = 2 h (* is the DPDS) (the hydrolyzed **9b** segment was not detected during the reaction).

5. Analysis of the Process of Peptidyl Selenoester-mediated *N*-linked Glycosylation Containing the Complex Undecasaccharide[4-6]

5.1. Synthesis of glycopeptide 10e

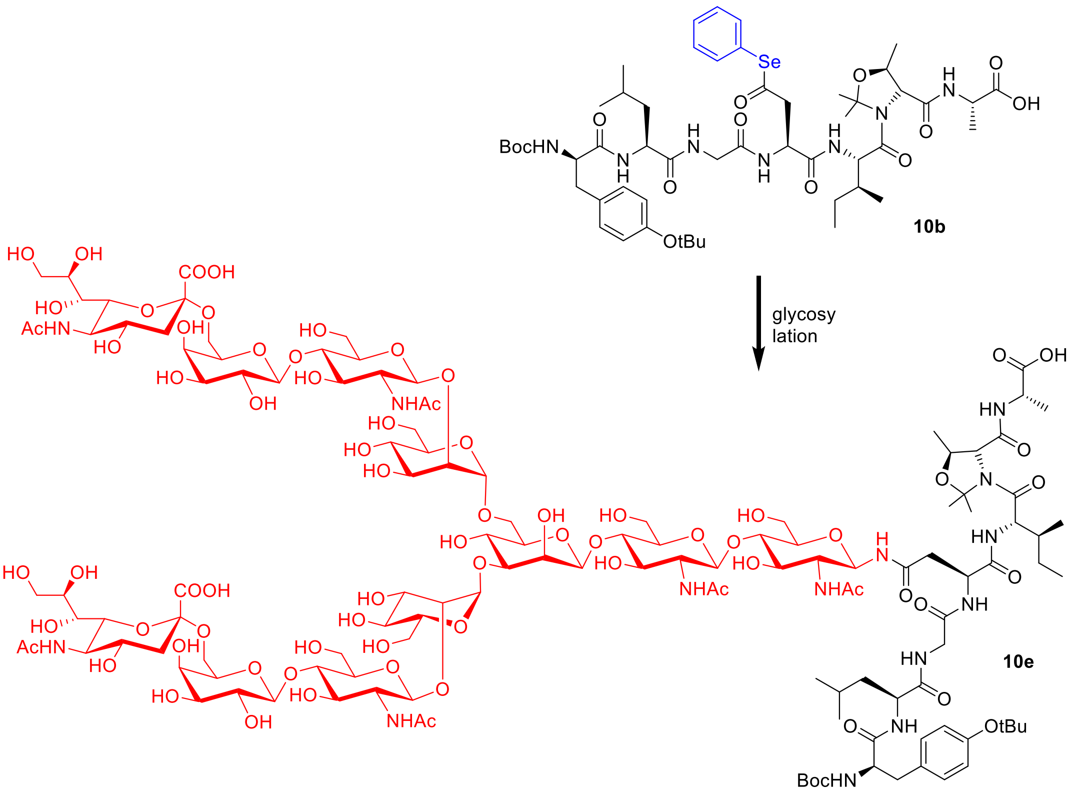

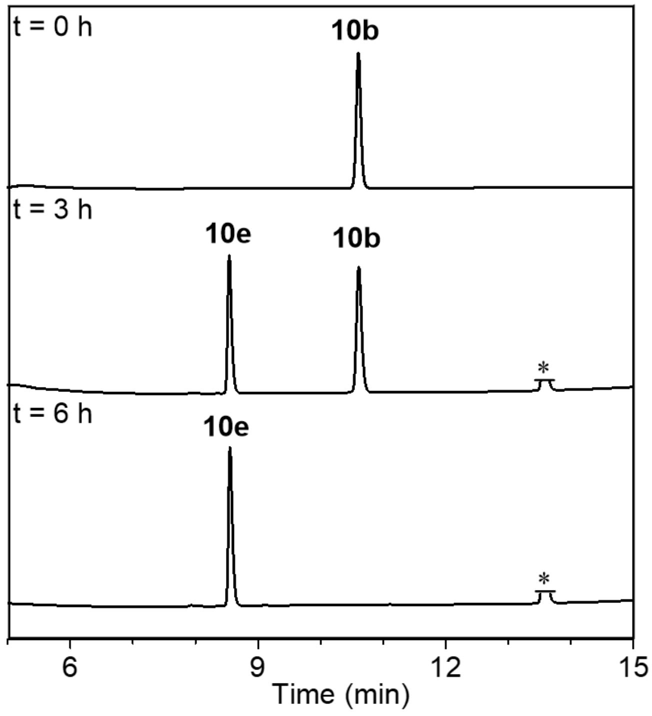

Figure S10. Analytical HPLC profiles of the ligation reaction (gradient: 80% to 100% CH_3CN over 15 min, λ = 254 nm): t = 0 h, t = 3 h, t = 6 h (* is the DPDS) (the hydrolyzed **10b** segment was not detected during the reaction).

5.2. Synthesis of glycopeptide 11e

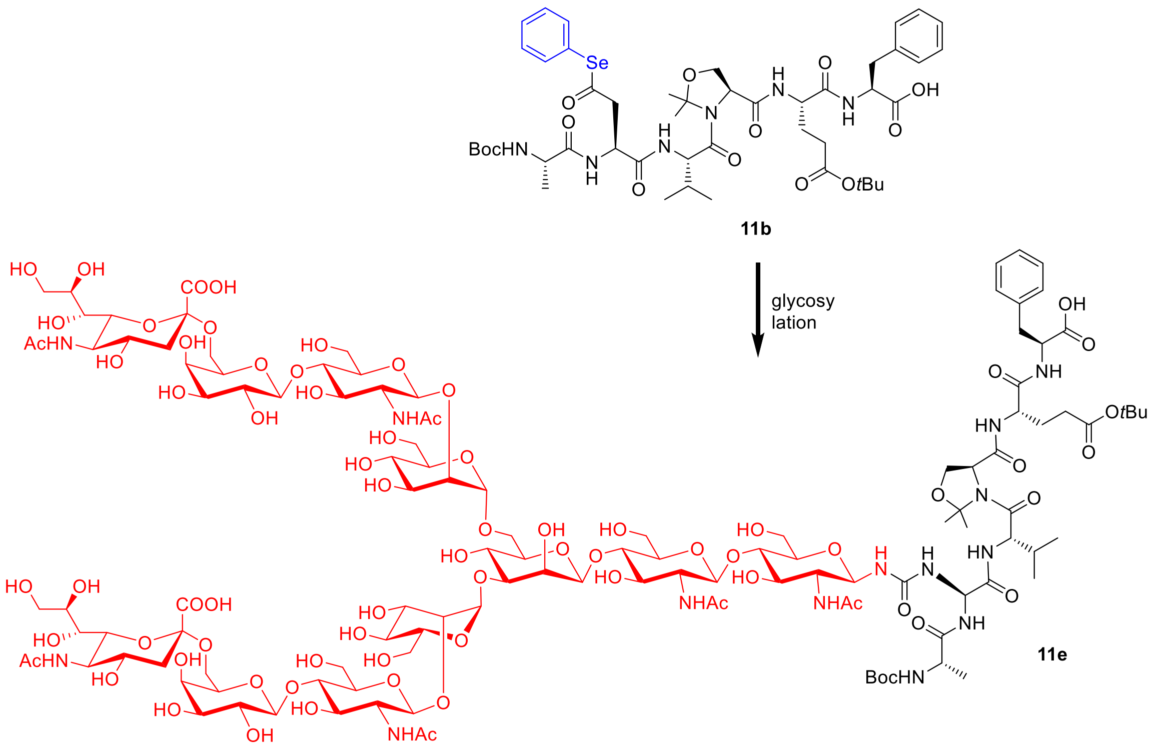

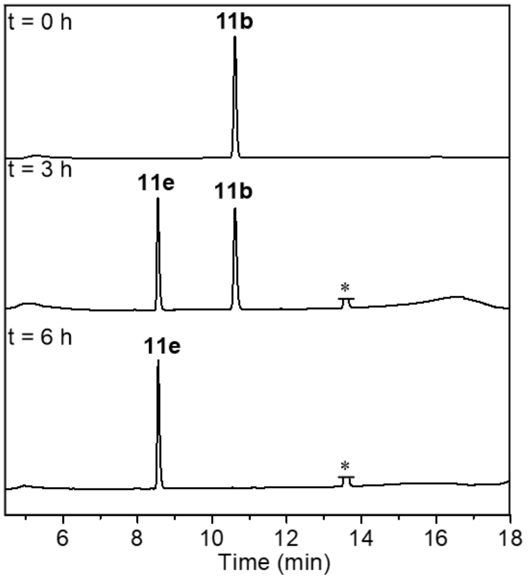

Figure S11. Analytical HPLC profiles of the ligation reaction (gradient: 80% to 100% CH_3CN over 18 min, λ = 254 nm): t = 0 h, t = 2 h, t = 4 h (* is the DPDS) (the hydrolyzed **11b** segment was not detected during the reaction).

5.3. Synthesis of glycopeptide 12e

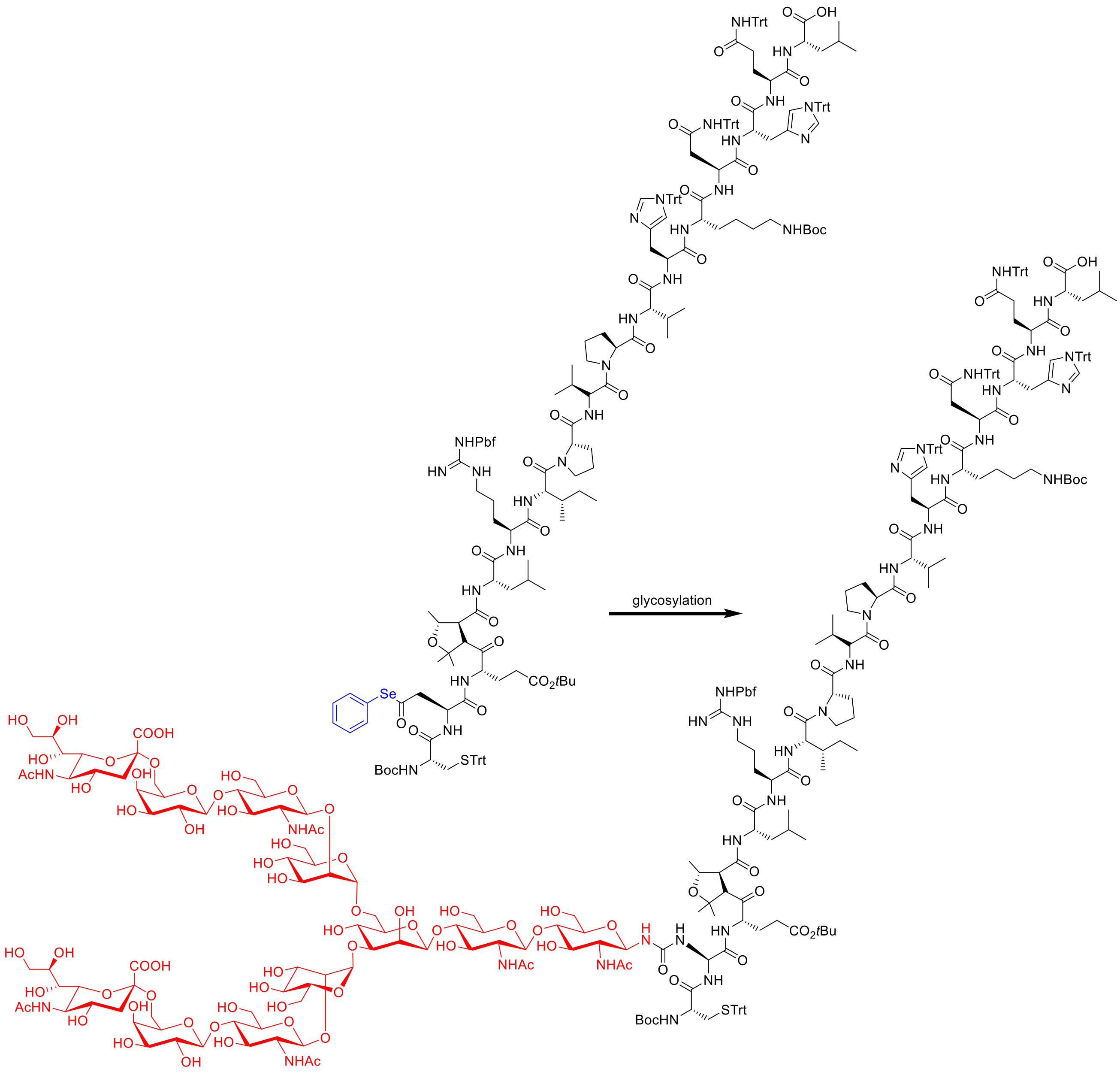

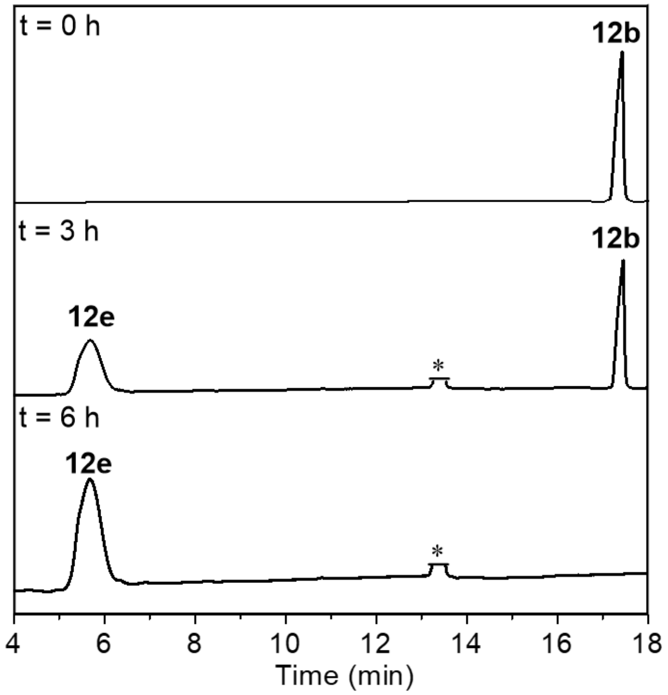

Figure S12. Analytical HPLC profiles of the ligation reaction (gradient: 80% to 100% CH_3CN over 18 min, 100% CH_3CN over 14-18 min, λ = 254 nm): t = 0 h, t = 3 h, t = 6 h (* is the DPDS) (the hydrolyzed **12b** segment was not detected during the reaction).

6. HPLC and ESI-MS spectra of peptidyl selenoesters

6.1. Peptidyl selenoester 4b

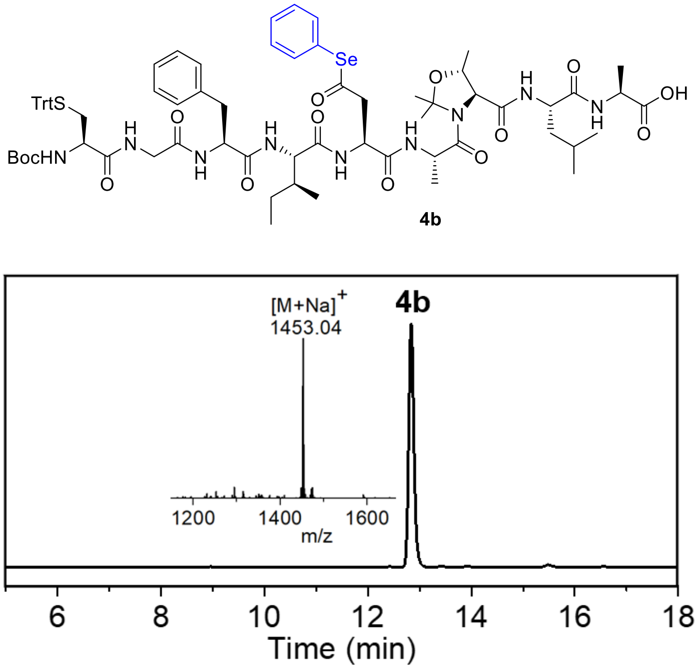

Figure S13. HPLC spectrum of purified selenoester **4b** (gradient: 80% to 100% CH_3CN over 18 min, λ = 254 nm). The isolated yield of **4b** by SPPS is about 80% (57 mg). ESI-MS calcd for **4b** $C_{73}H_{92}N_9O_{14}SSe$ $[M+Na]^+$ *m/z* = 1453.57; observed: $[M+Na]^+$ *m/z* = 1453.04.

6.2. Peptidyl selenoester 5b

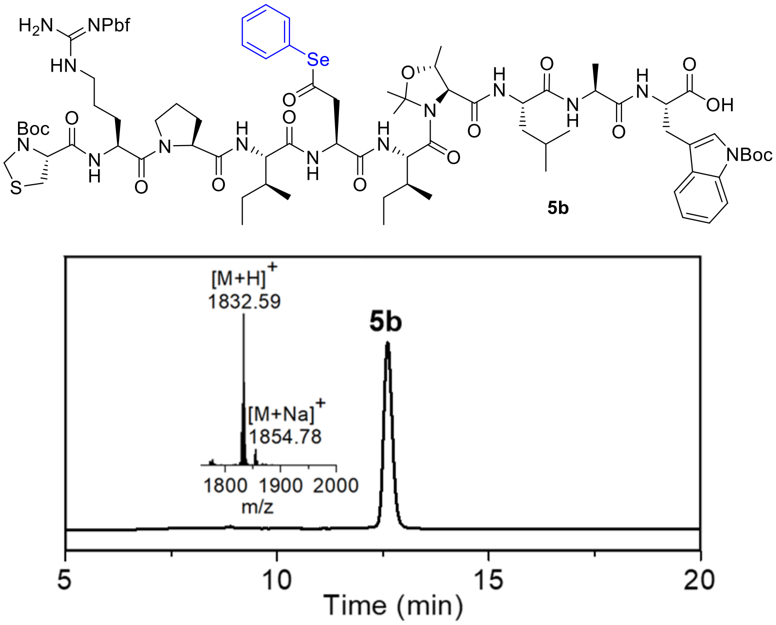

Figure S14. HPLC spectrum of purified selenoester **5b** (gradient: 90% CH_3CN over 20 min, λ = 254 nm). The isolated yield of **5b** by SPPS is about 83% (77 mg). ESI-MS calcd for **5b** $C_{87}H_{126}N_{14}O_{20}S2Se$ $[M+H]^+$ *m/z* = 1832.12, $[M+Na]^+$ *m/z* = 1854.12; observed: $[M+H]^+$ *m/z* = 1832.59, $[M+Na]^+$ *m/z* = 1854.78.

6.3. Peptidyl selenoester 6b

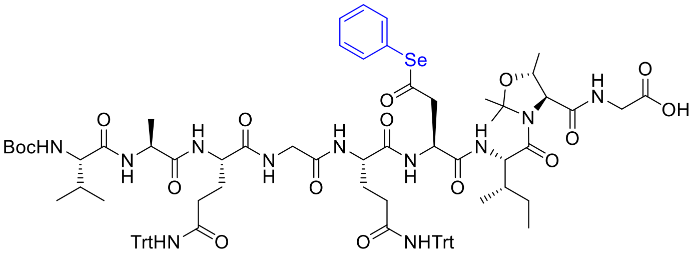

6b

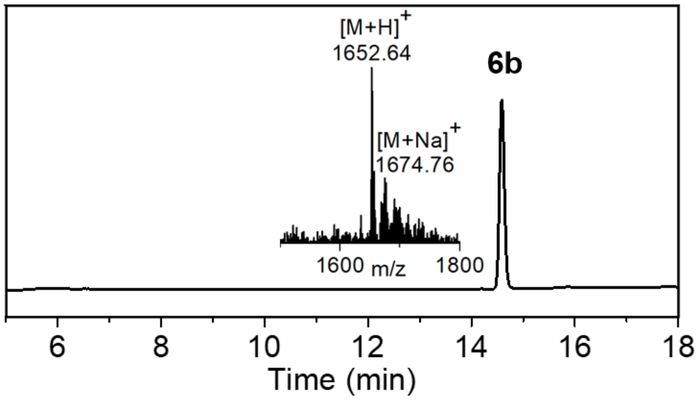

Figure S15. HPLC spectrum of purified selenoester **6b** (gradient: 75% to 100% CH_3CN over 18 min, λ = 254 nm). The isolated yield of **6b** by SPPS is about 75% (62 mg). ESI-MS calcd for **6b** $C_{88}H_{105}N_{11}O_{16}Se$ $[M+H]^+$ *m/z* = 1652.69, $[M+Na]^+$ *m/z* = 1674.69; observed: $[M+H]^+$ *m/z* = 1652.64, $[M+Na]^+$ *m/z* = 1674.76.

6.4. Peptidyl selenoester 7b

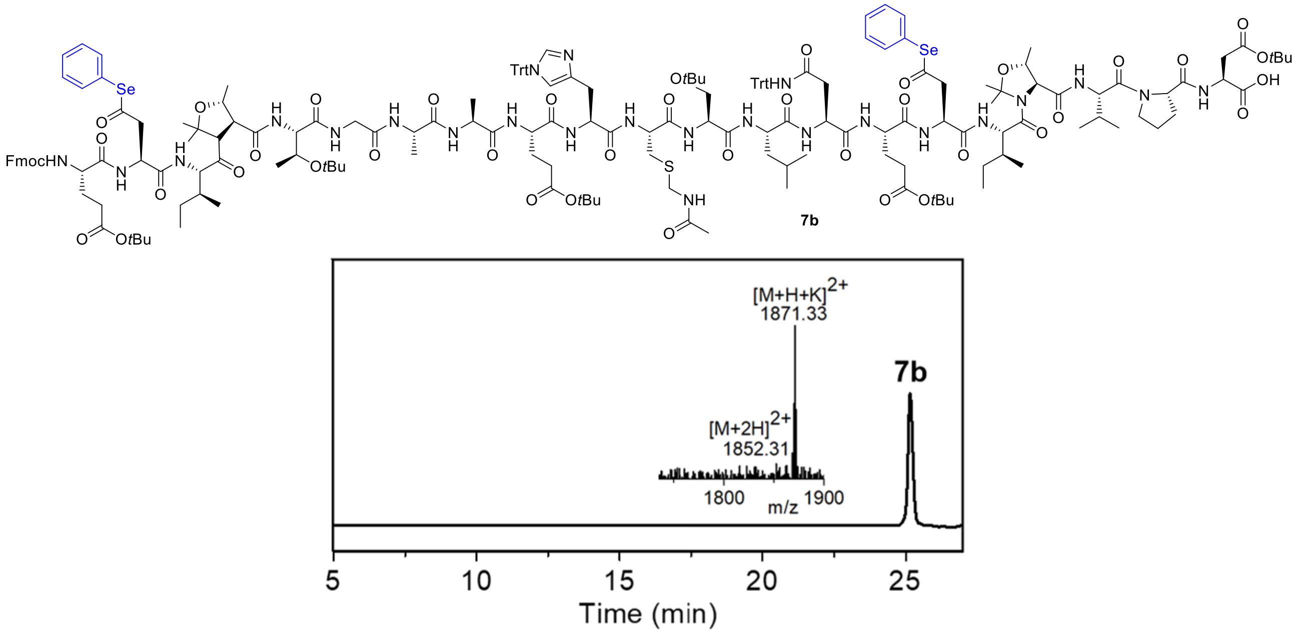

Figure S16. HPLC spectrum of purified selenoester **7b** (gradient: 50% to 100% CH_3CN over 27 min, λ = 254 nm). The isolated yield of **7b** by SPPS is about 67% (124 mg). ESI-MS calcd for **7b** $C_{190}H_{252}N_{24}O_{40}SSe_2$ $[M+2H]^{2+}$ *m/z* = 1852.11, $[M+H+K]^{2+}$ *m/z* = 1871.11; observed: $[M+2H]^{2+}$ *m/z* = 1852.31, $[M+H+K]^{2+}$ *m/z* = 1871.33.

6.5. Peptidyl selenoester 8b

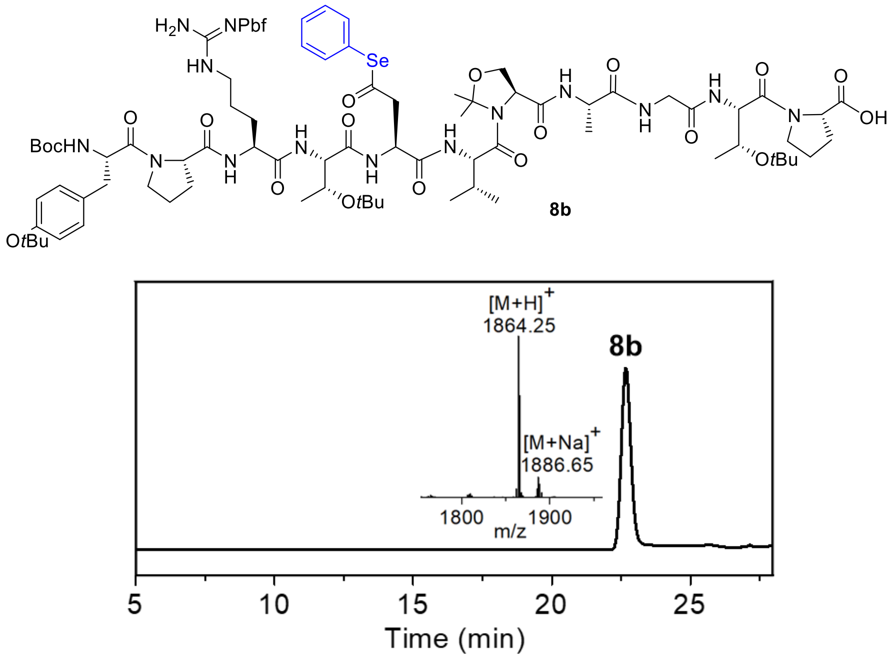

Figure S17. HPLC spectrum of purified selenoester **8b** (gradient: 50% to 100% CH_3CN over 27 min, λ = 254 nm). The isolated yield of **8b** by SPPS is about 72% (67 mg). ESI-MS calcd for **8b** $C_{89}H_{134}N_{14}O_{22}SSe$ $[M+H]^+$ *m/z* = 1864.14, $[M+Na]^+$ *m/z* = 1886.14; observed: $[M+H]^+$ *m/z* = 1864.25, $[M+Na]^+$ *m/z* = 1886.65.

6.6. Peptidyl selenoester 9b

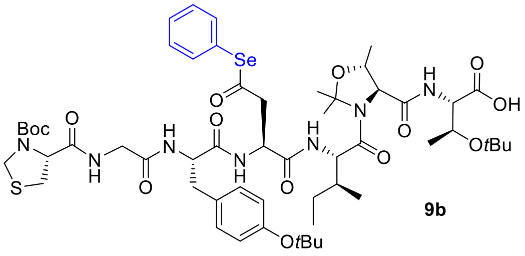

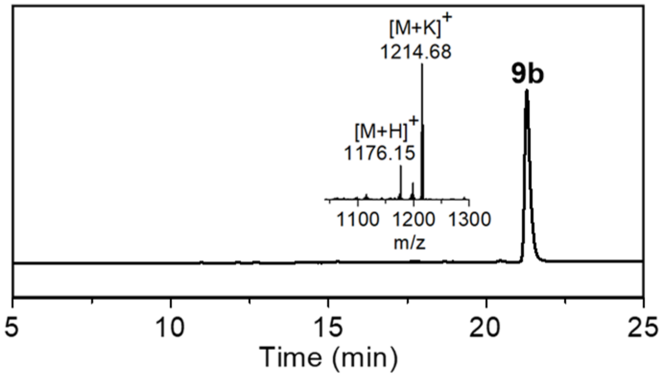

Figure S18. HPLC spectrum of purified selenoester **9b** (gradient: 35% to 100% CH_3CN over 25 min, λ = 254 nm). The isolated yield of **9b** by SPPS is about 81% (67 mg). ESI-MS calcd for **9b** $C_{55}H_{81}N_7O_{14}SSe$ $[M+H]^+$ *m/z* = 1176.47, $[M+K]^+$ *m/z* = 1214.47; observed: $[M+H]^+$ *m/z* = 1176.15, $[M+K]^+$ *m/z* = 1214.68.

6.7. Peptidyl selenoester 10b

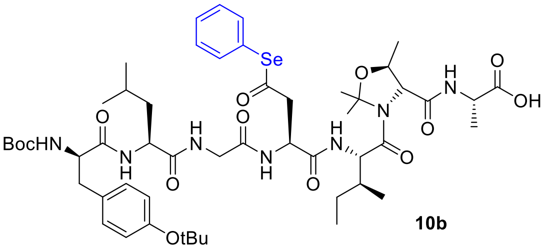

10b

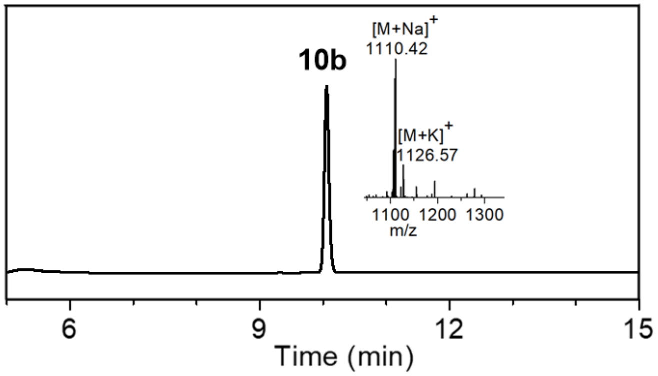

Figure S19. HPLC spectrum of purified selenoester **10b** (gradient: 80% to 100% CH_3CN over 15 min, λ = 254 nm). The isolated yield of **10b** by SPPS is about 77% (42 mg). ESI-MS calcd for **10b** $C_{52}H_{77}N_7O_{13}Se$ $[M+Na]^+$ m/z = 1110.47, $[M+K]^+$ m/z = 1126.47; observed: $[M+Na]^+$ m/z = 1110.42, $[M+K]^+$ m/z = 1126.57.

6.8. Peptidyl selenoester 11b

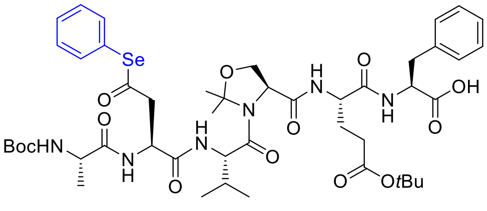

11b

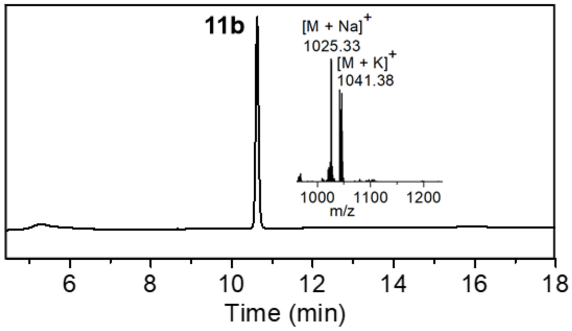

Figure S20. HPLC spectrum of purified selenoester **11b** (gradient: 80% to 100% CH_3CN over 18 min, λ = 254 nm). The isolated yield of **11b** by SPPS is about 79% (40 mg). ESI-MS calcd for **11b** $C_{47}H_{66}N_6O_{13}Se$ $[M+Na]^+$ *m/z* = 1025.38, $[M+K]^+$ *m/z* = 1041.38; observed: $[M+H]^+$ *m/z* = 1025.33, $[M+Na]^+$ *m/z* = 1041.38.

6.9. Peptidyl selenoester 12b

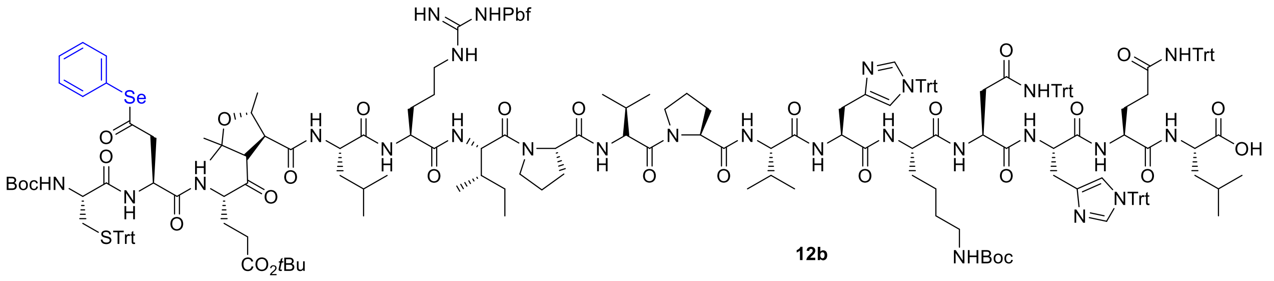

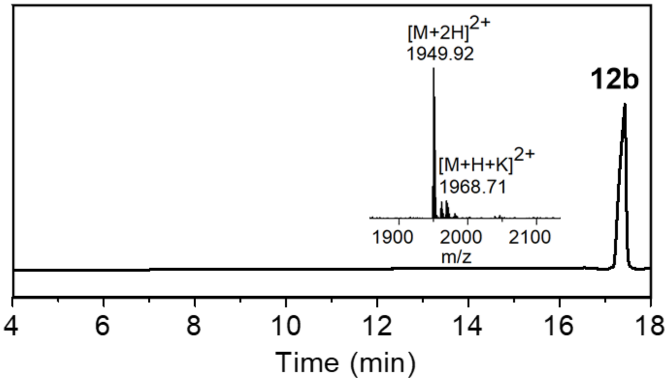

Figure S21. HPLC spectrum of purified selenoester **12b** (gradient: 80% to 100% CH_3CN over 18 min, 100% CH_3CN over 14-18 min, λ = 254 nm). The isolated yield of **12b** by SPPS is about 58% (113 mg). ESI-MS calcd for **12b** $C_{218}H_{261}N_{27}O_{31}S_2Se$ $[M+2H]^{2+}$ *m/z* = 1949.92, $[M+H+K]^{2+}$ *m/z* = 1968.91; observed: $[M+2H]^{2+}$ *m/z* = 1949.92, $[M+H+K]^{2+}$ *m/z* = 1968.71.

7. HPLC and ESI-MS Spectra of *N*-linked Glycopeptides

7.1. Glycopeptide 4c

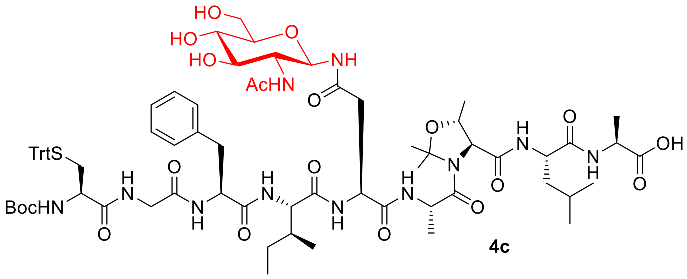

4c

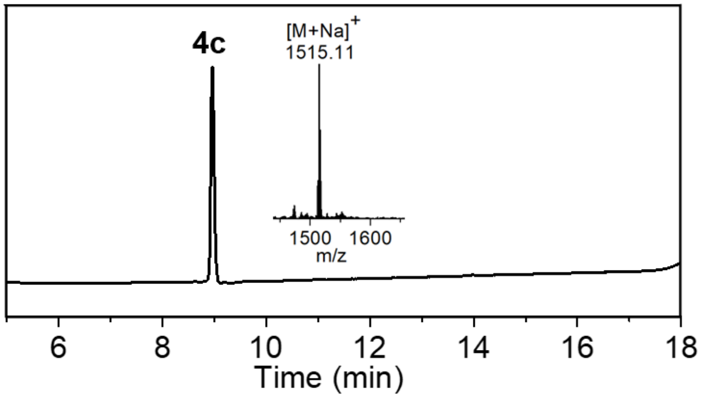

Figure S22. HPLC spectrum of purified glycopeptide **4c** (gradient: 80% to 100% CH_3CN over 18 min, λ = 254 nm). The isolated yield of **4c** is about 78% (6 mg, in solution) and 67% (50 mg, on resin), ESI-MS calcd for **4c** $C_{75}H_{102}N_{11}O_{19}S$ $[M+Na]^+$ *m/z* = 1515.71; observed: $[M+Na]^+$ *m/z* = 1515.11.

7.2. Glycopeptide 5c

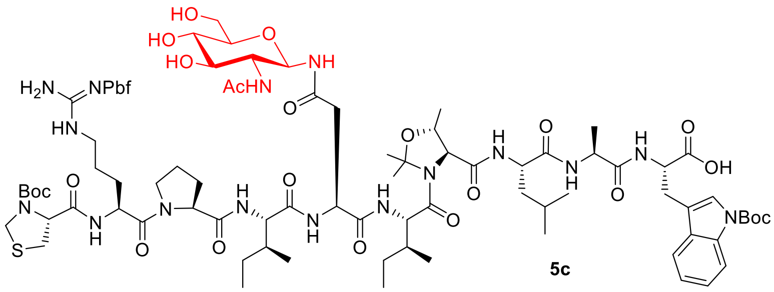

5c

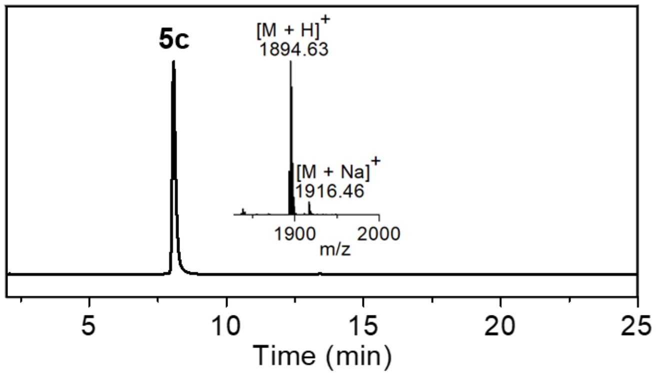

Figure S23. HPLC spectrum of purified glycopeptide **5c** (gradient: 90% CH_3CN over 25 min, λ = 254 nm). The isolated yield of **5c** is about 83% (8 mg, in solution) and 65% (61 mg, on resin), ESI-MS calcd for **5c** $C_{89}H_{136}N_{16}O_{25}S_2$ $[M+H]^+$ *m/z* = 1894.93, $[M+Na]^+$ *m/z* = 1916.93; observed: $[M+H]^+$ *m/z* = 1894.63, $[M+Na]^+$ *m/z* = 1916.46.

7.3. Glycopeptide 6c

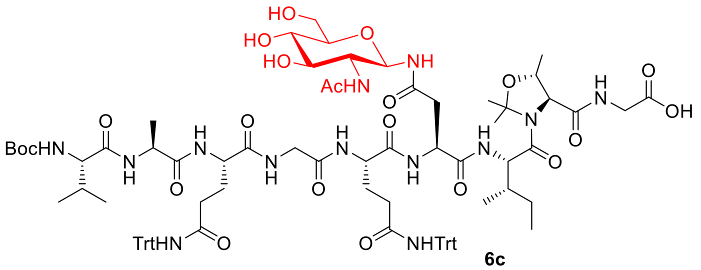

6c

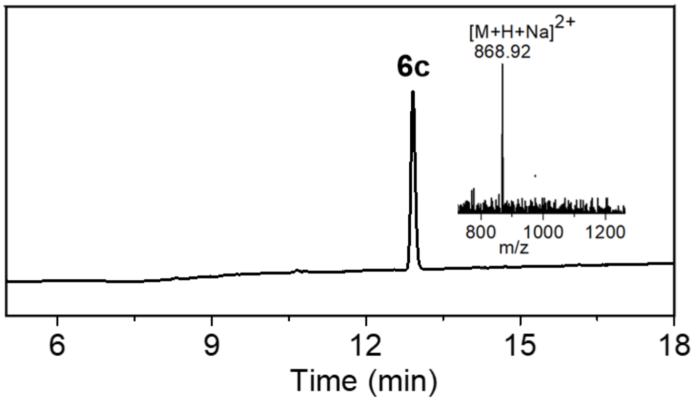

Figure S25. HPLC spectrum of purified glycopeptide **6c** (gradient: 75% to 100% CH_3CN over 18 min, λ = 254 nm). The isolated yield of **6c** is about 69% (6 mg), ESI-MS calcd for **6c** $C_{75}H_{102}N_{11}O_{19}S$ $[M+H+Na]^{2+}$ *m/z* = 868.91; observed: $[M+H+Na]^{2+}$ *m/z* = 868.92.

7.4. Glycopeptide 7c

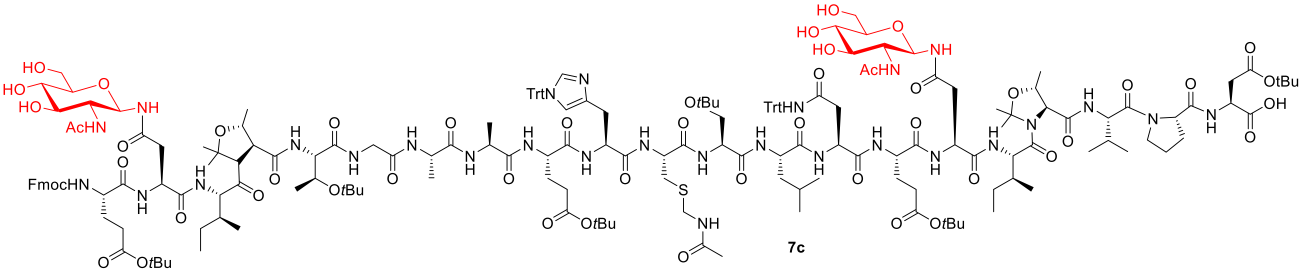

7c

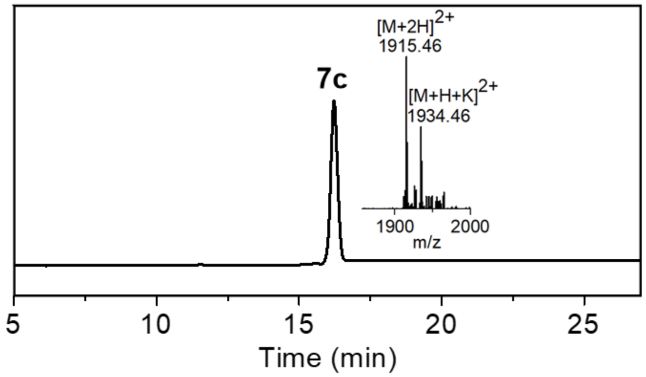

Figure S26. HPLC spectrum of purified glycopeptide **7c** (gradient: 50% to 100% CH_3CN over 27 min, λ = 254 nm). The isolated yield of **7c** is about 79% (15 mg, in solution). ESI-MS calcd for **11c** $C_{194}H_{272}N_{28}O_{50}S$ $[M+2H]^{2+}$ *m/z* = 1915.26, $[M+H+K]^{2+}$ *m/z* = 1934.26; observed: $[M+2H]^{2+}$ *m/z* = 1915.46, $[M+H+K]^{2+}$ *m/z* = 1934.46.

7.5. Glycopeptide 5d

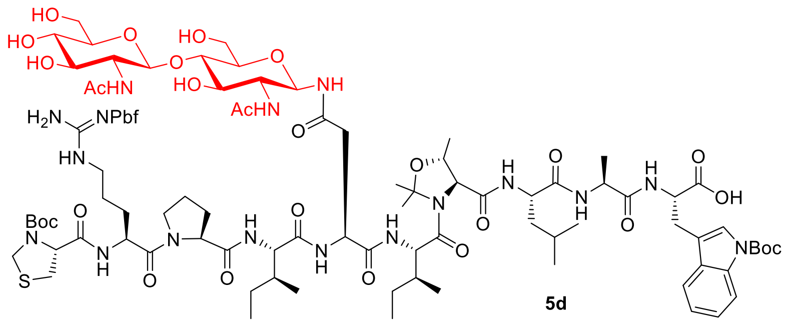

5d

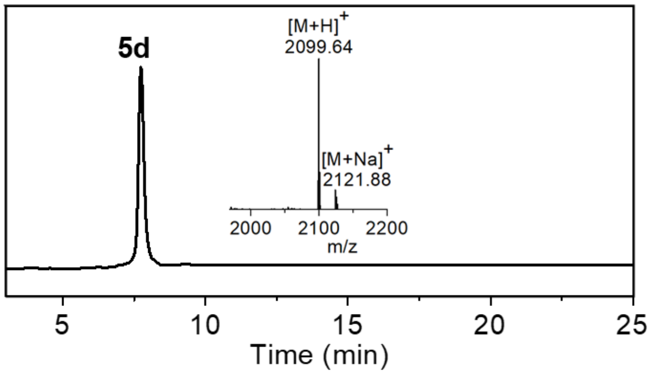

Figure S24. HPLC spectrum of purified glycopeptide **5d** (gradient: 90% CH_3CN over 25 min, λ = 254 nm). The isolated yield of **5d** is about 79% (8 mg, in solution). ESI-MS calcd for **5d** $C_{97}H_{151}N_{17}O_{30}S_2$ $[M+H]^+$ *m/z* = 2099.46, $[M+Na]^+$ *m/z* = 2121.46; observed: $[M+H]^+$ *m/z* = 2099.64, $[M+Na]^+$ *m/z* = 2121.88.

7.6. Glycopeptide 8d

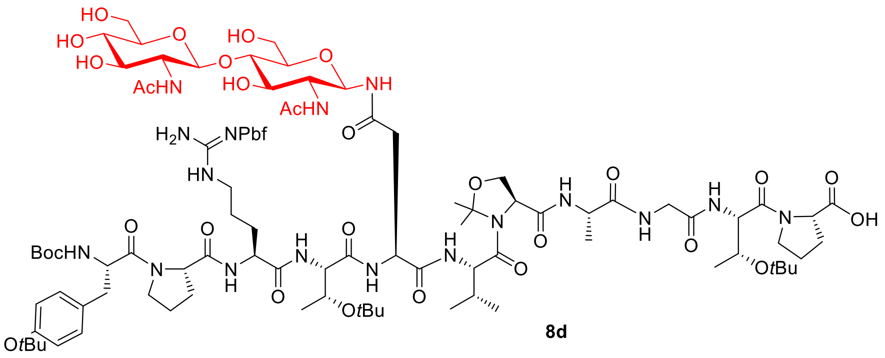

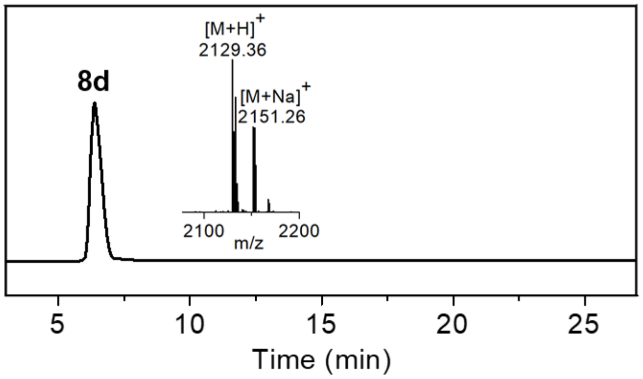

Figure S27. HPLC spectrum of purified glycopeptide **8d** (gradient: 50% to 100% CH_3CN over 27 min, λ = 254 nm). The isolated yield of **8d** is about 82% (8 mg). ESI-MS calcd for **8d** $C_{99}H_{157}N_{17}O_{32}S$ $[M+H]^+$ *m/z* = 2129.09, $[M+Na]^+$ *m/z* = 2151.09; observed: $[M+H]^+$ *m/z* = 2129.36, $[M+Na]^+$ *m/z* = 2151.26.

7.7. Glycopeptide 9d

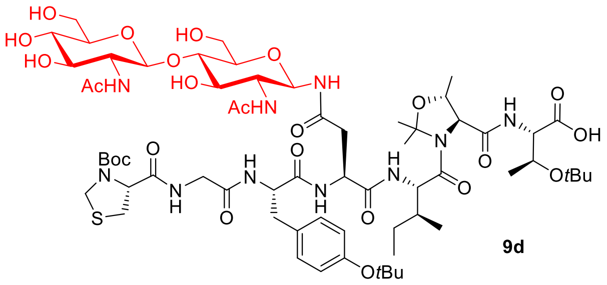

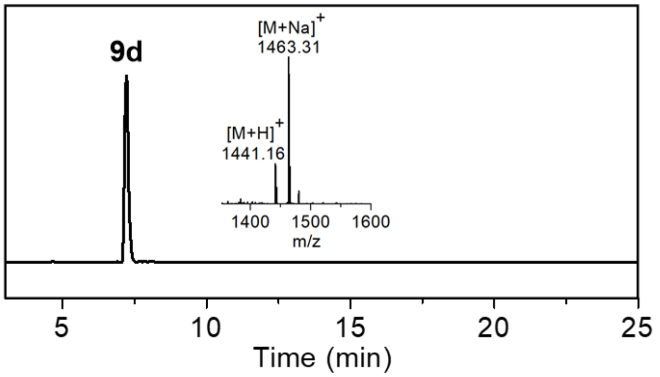

Figure S28. HPLC spectrum of purified glycopeptide **9d** (gradient: 85% to 100% CH_3CN over 25 min, λ = 254 nm). The isolated yield of **9d** is about 84% (6 mg). ESI-MS calcd for **9d** $C_{65}H_{104}N_{10}O_{24}S$ $[M+H]^+$ *m/z* = 1441.69, $[M+Na]^+$ *m/z* = 1463.69; observed: $[M+H]^+$ *m/z* = 1441.16, $[M+Na]^+$ *m/z* = 1463.31.

7.8. Glycopeptide 10e

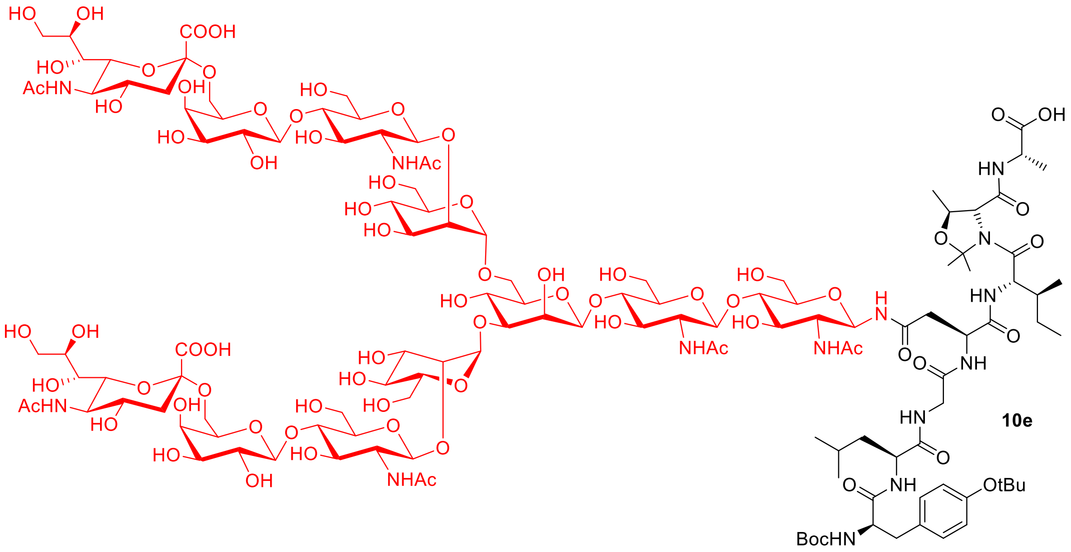

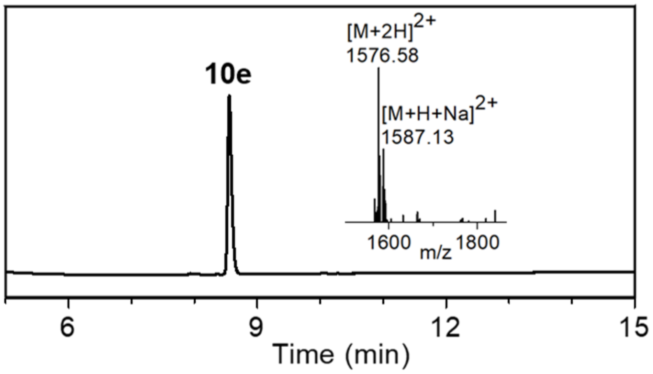

Figure S29. HPLC spectrum of purified glycopeptide **10e** (gradient: 80% to 100% CH_3CN over 15 min, λ = 254 nm). The isolated yield of **10e** is about 59% (4 mg). ESI-MS calcd for **10e** $C_{130}H_{210}N_{14}O_{74}$ $[M+2H]^{2+}$ *m/z* = 1576.66, $[M+H+Na]^{2+}$ *m/z* = 1587.66; observed: $[M+2H]^{2+}$ *m/z* = 1576.58, $[M+H+Na]^{2+}$ *m/z* = 1587.13.

7.9. Glycopeptide 11e

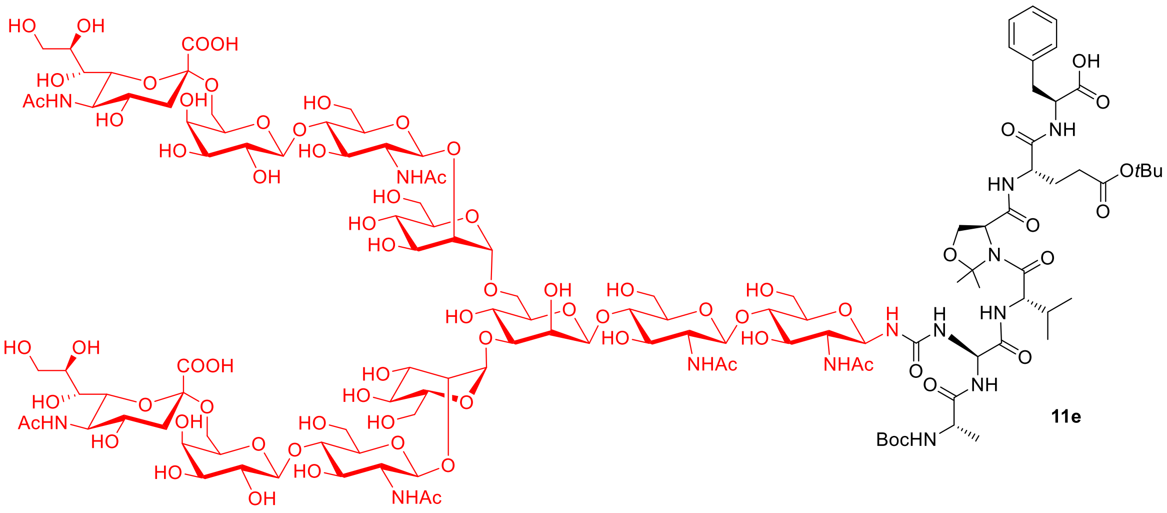

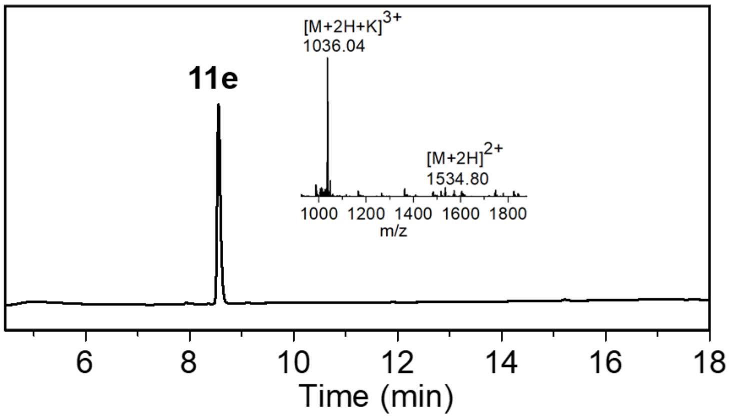

Figure S30. HPLC spectrum of purified glycopeptide **11e** (gradient: 80% to 100% CH_3CN over 18 min, λ = 254 nm). The isolated yield of **11e** is about 68% (4 mg). ESI-MS calcd for **11e** $C_{124}H_{198}N_{14}O_{74}$ $[M+2H]^{2+}$ *m/z* = 1534.61, $[M+2H+K]^{3+}$ *m/z* = 1036.07; observed: $[M+2H]^{2+}$ *m/z* = 1534.80, $[M+2H+K]^{3+}$ *m/z* = 1036.04.

7.10. Glycopeptide 12e

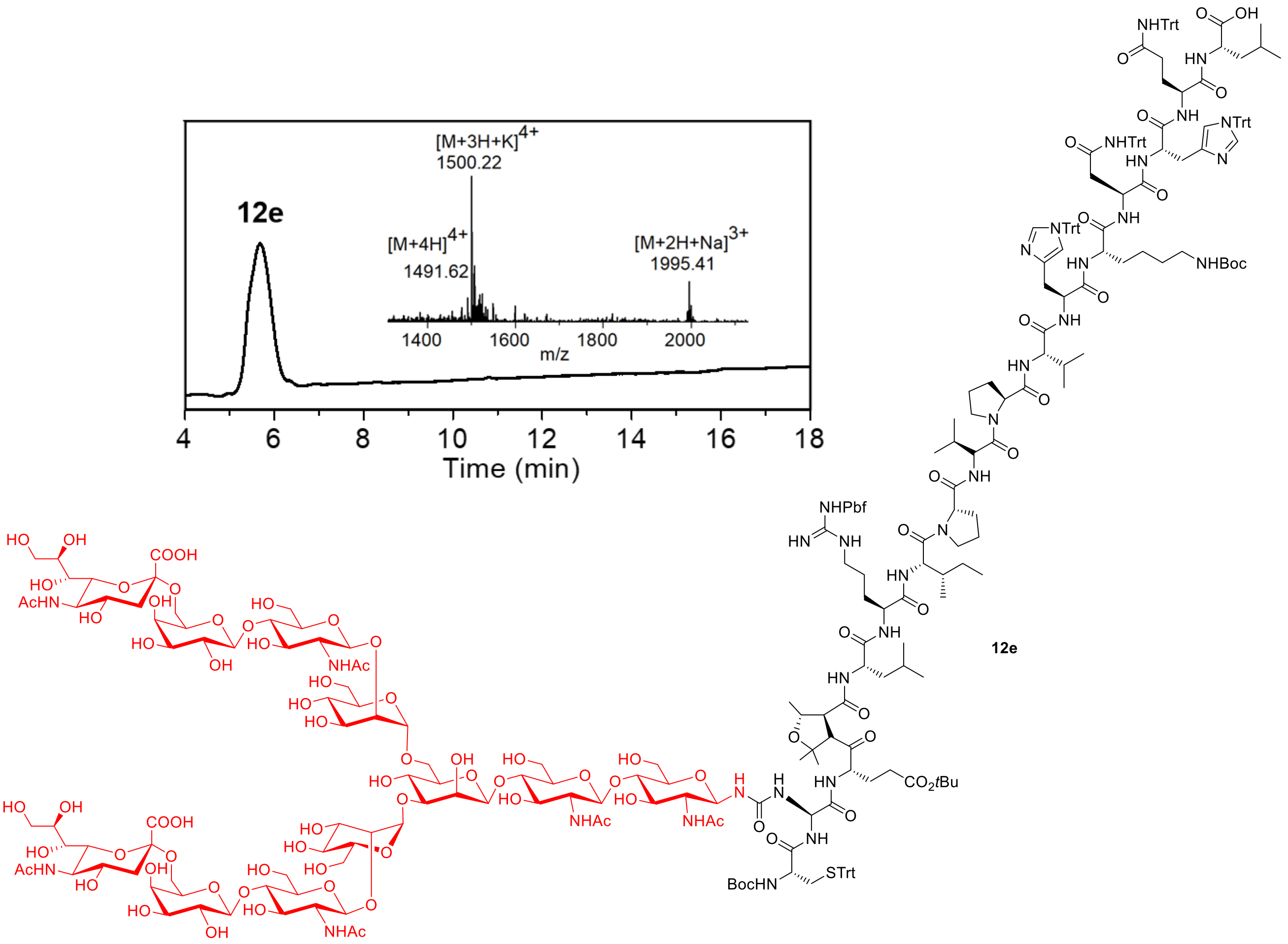

Figure S31. HPLC spectrum of purified glycopeptide **12e** (gradient: 80% to 100% CH_3CN over 14 min, 100% CH_3CN over 14-18 min, λ = 254 nm). The isolated yield of **12e** is about 62% (7 mg, in solution). ESI-MS calcd for **12e** $C_{297}H_{395}N_{33}O_{92}S_2$ $[M+4H]^{4+}$ *m/z* = 1491.17, $[M+3H+K]^{4+}$ *m/z* = 1500.66, $[M+2H+Na]^{3+}$ *m/z* = 1995.22; observed: $[M+4H]^{4+}$ *m/z* = 1491.62, $[M+3H+K]^{4+}$ *m/z* = 1500.22. $[M+2H+Na]^{3+}$ *m/z* = 1995.41.

8. ^{1}H NMR and ^{13}C NMR Spectra

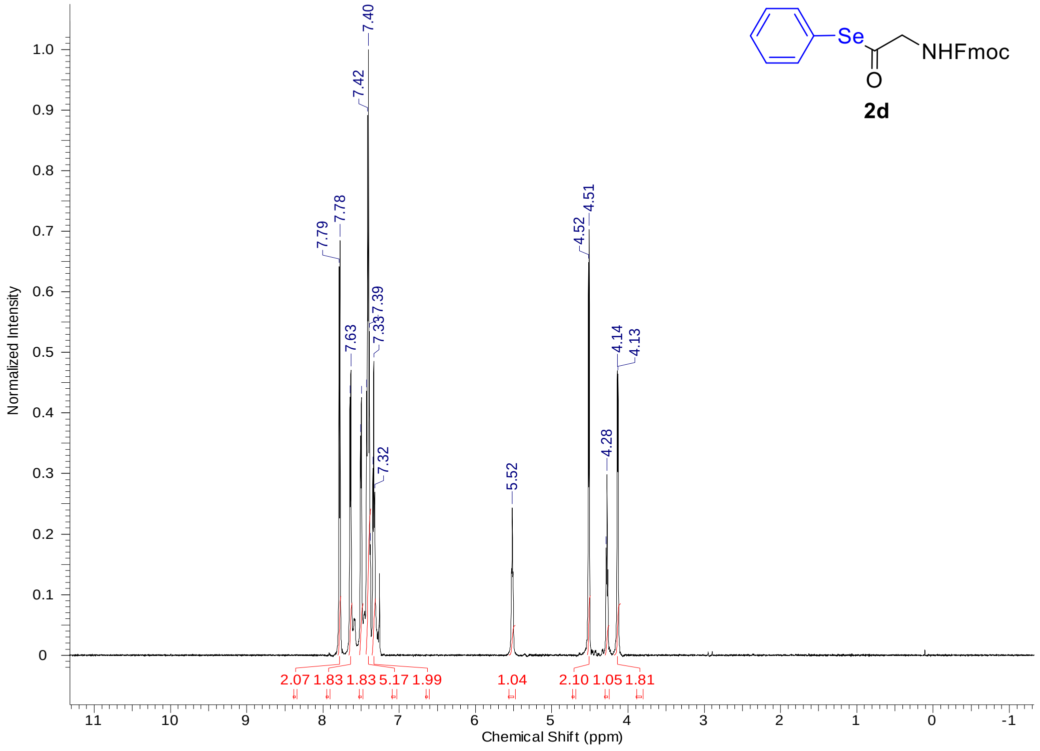

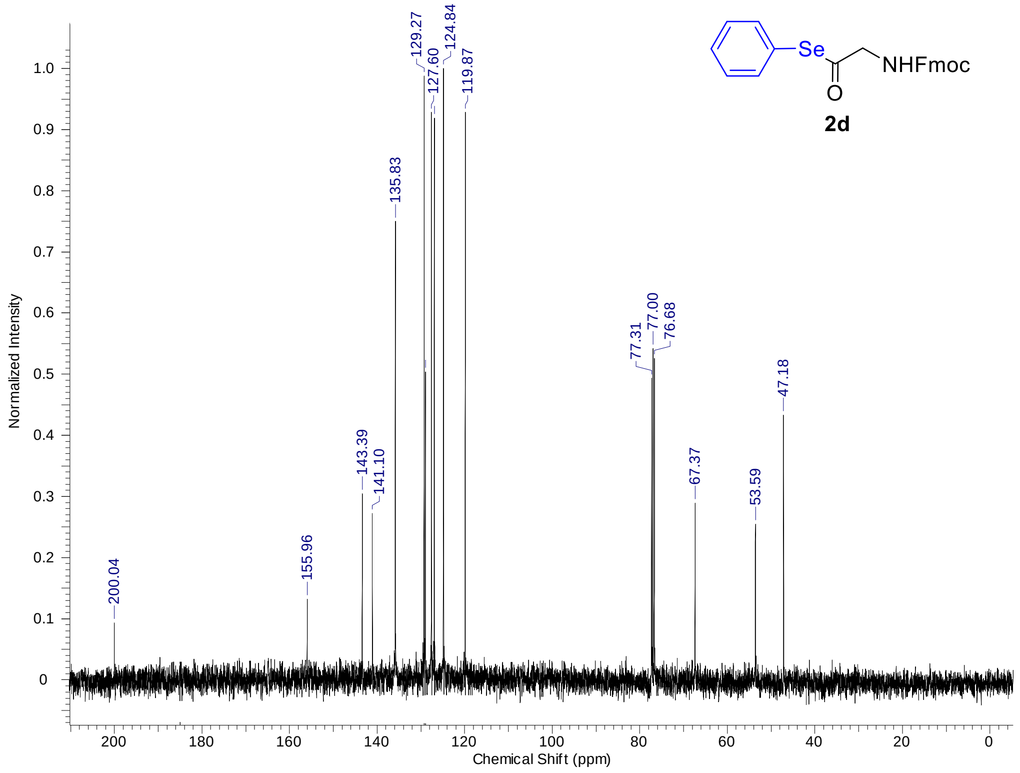

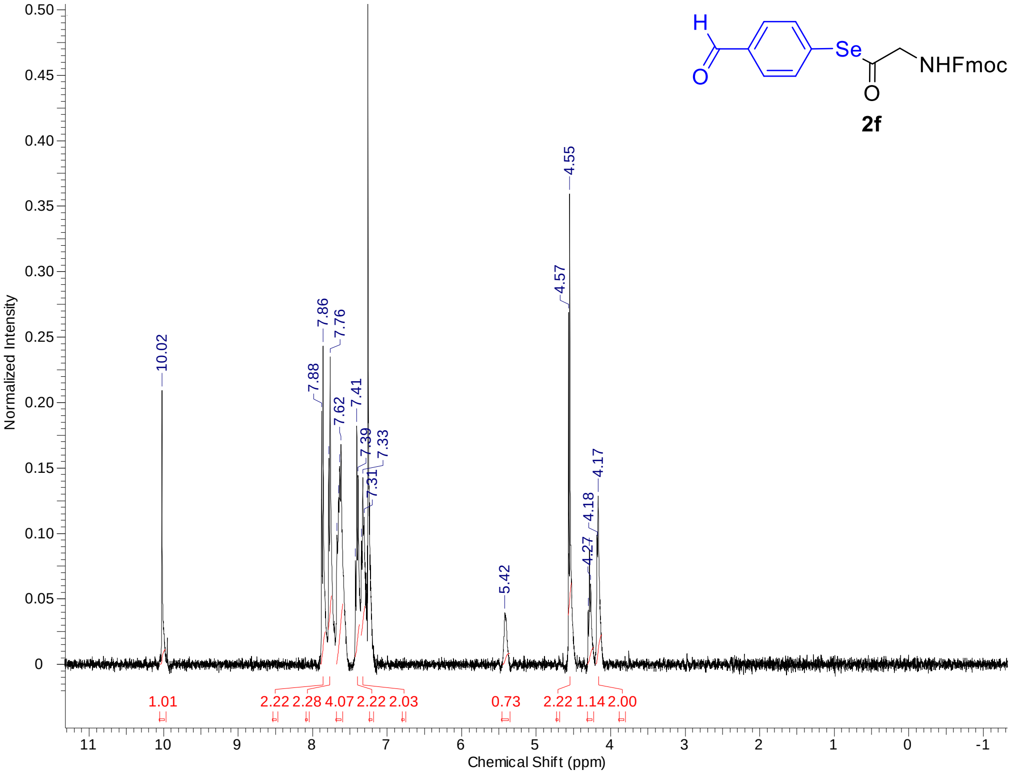

Se
NHFmoc
O
H
O
2f
10.02
7.88
7.86
7.76
7.62
7.41
7.39
7.33
7.31
5.42
4.57
4.55
4.27
4.18
4.17
1.01
2.22
2.28
4.07
2.22
2.03
0.73
2.22
1.14
2.00
Normalized Intensity
Chemical Shift (ppm)

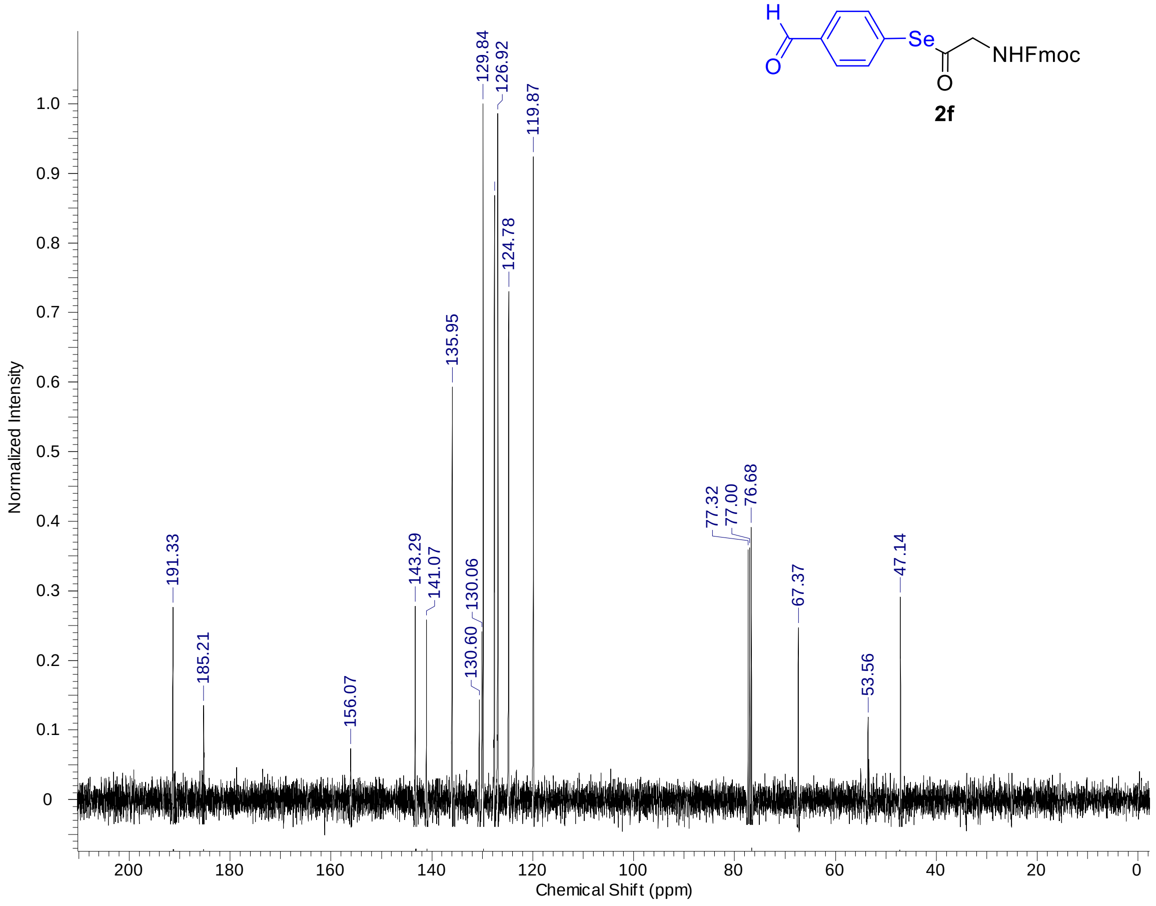

Se
NHFmoc
O
H
O
2f
191.33
185.21
156.07
143.29
141.07
135.95
130.60
130.06
129.84
126.92
124.78
119.87
77.32
77.00
76.68
67.37
53.56
47.14
Normalized Intensity
Chemical Shift (ppm)

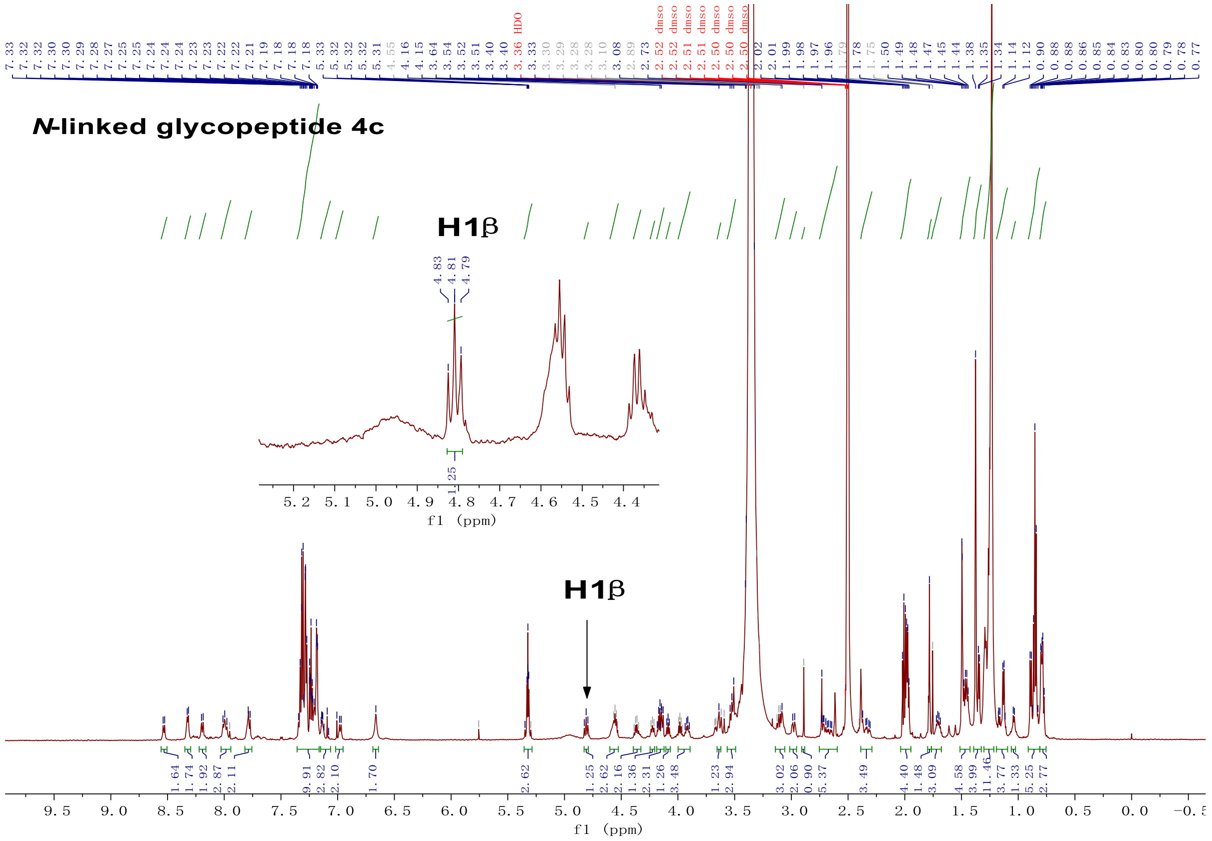

9. Supplementary References

[1] Du, J.-J., Gao, X.-F., Xin, L.-M., Lei, Z., Liu, Z., and Guo, J. (2016). Convergent Synthesis of N-Linked Glycopeptides via Aminolysis of *ω*-Asp *p*-Nitrophenyl Thioesters in Solution. *Org. Lett.* 18, 4828-4831. doi: 10.1021/acs.orglett.6b02288

[2] Raj, M., Wu, H., Blosser, S. L., Vittoria, M. A., and Arora, P. S. (2015). Aldehyde Capture Ligation for Synthesis of Native Peptide Bonds. *J. Am. Chem. Soc.* 137, 6932–6940. doi: 10.1021/jacs.5b03538

[3] Likhosherstov, L.M., Novikova, O.S., Derevitskaja, V.A., and Kochetkov, N.K. (1986). A new simple synthesis of amino sugar *β*-D-glycosylamines. *Carbohydr. Res.* 146, C1-C5. doi: 10.1016/0008-6215(86)85037-6

[4] Seko, A., Koketsu, M., Nishizono, M., Enoki, Y., Ibrahim, H.R., Juneja, L.R., Kim, M., and Yamamoto, T. (1997). Occurrence of a sialylglycopeptide and free sialylglycans in hen's egg yolk. *Biochim. Biophys. Acta Gen. Subj.* 1335, 23-32. doi: 10.1016/S0304-4165(96)00118-3

[5] Zou, Y., Wu, Z., Chen, L., Liu, X., Gu, G., Xue, M., Wang, P.G., and Chen, M. (2012). An efficient approach for large-scale production of sialyglycopeptides from egg yolks. *J. Carbohydr. Chem.* 31, 436-446. doi: 10.1080/07328303.2012.666689

[6] Sun, B., Bao, W., Tian, X., Li, M., Liu, H., Dong, J., and Huang, W. (2014). A simplified procedure for gram-scale production of sialylglycopeptide (SGP) from egg yolks and subsequent semi-synthesis of Man3GlcNAc oxazoline. *Carbohydr. Res.* 396, 62-69. doi: 10.1016/j.carres.2014.07.013
